# Development of a Novel Class of Pyridazinone Derivatives as Selective MAO-B Inhibitors

**DOI:** 10.3390/molecules27123801

**Published:** 2022-06-13

**Authors:** Mehmet Abdullah Alagöz, Jong Min Oh, Yaren Nur Zenni, Zeynep Özdemir, Mohamed A. Abdelgawad, Ibrahim A. Naguib, Mohammed M. Ghoneim, Nicola Gambacorta, Orazio Nicolotti, Hoon Kim, Bijo Mathew

**Affiliations:** 1 Department of Pharmaceutical Chemistry, Faculty of Pharmacy, Inonu University, 44280 Malatya, Turkey; mehmet.alagoz@inonu.edu.tr (M.A.A.); yarennur.zenni@inonu.edu.tr (Y.N.Z.); zeynep.bulut@inonu.edu.tr (Z.Ö.); 2 Department of Pharmacy, and Research Institute of Life Pharmaceutical Sciences, Sunchon National University, Suncheon 57922, Korea; ddazzo005@naver.com; 3 Department of Pharmaceutical Chemistry, College of Pharmacy, Jouf University, Sakaka, Al Jouf 72341, Saudi Arabia; mhmdgwd@ju.edu.sa; 4 Department of Pharmaceutical Chemistry, College of Pharmacy, Taif University, P.O. Box 11099, Taif 21944, Saudi Arabia; i.abdelaal@tu.edu.sa; 5 Department of Pharmacy Practice, Faculty of Pharmacy, AlMaarefa University, Ad Diriyah 13713, Saudi Arabia; mghoneim@mcst.edu.sa; 6 Dipartimento di Farmacia—Scienze del Farmaco, Università degli Studi di Bari “Aldo Moro”, Via E. Orabona, 4, 70125 Bari, Italy; nicola.gambacorta1@uniba.it (N.G.); orazio.nicolotti@uniba.it (O.N.); 7 Department of Pharmaceutical Chemistry, Amrita School of Pharmacy, Amrita Vishwa Vidyapeetham, AIMS Health Sciences Campus, Kochi 682 041, India

**Keywords:** pyridazinones, monoamine oxidase-B, kinetics, reversibility, PAMPA, docking

## Abstract

Sixteen compounds (**TR1–TR16**) were synthesized and evaluated for their inhibitory activities against monoamine oxidase A and B (MAOs). Most of the derivatives showed potent and highly selective MAO-B inhibition. Compound **TR16** was the most potent inhibitor against MAO-B with an IC_50_ value of 0.17 μM, followed by **TR2** (IC_50_ = 0.27 μM). **TR2** and **TR16** selectivity index (SI) values for MAO-B versus MAO-A were 84.96 and higher than 235.29, respectively. Compared to the basic structures, the *para*-chloro substituent in **TR2** and **TR16** increased the inhibitory activity of MAO-B. **TR2** and **TR16** were reversible MAO-B inhibitors that were competitive, with K_i_ values of 0.230 ± 0.004 and 0.149 ± 0.016 µM, respectively. The PAMPA method indicated that compounds **TR2** and **TR16** had the tendency to traverse the blood–brain barrier. Docking investigations revealed that lead compounds were beneficial for MAO-B inhibition via association with key as well as selective E84 or Y326 residues, but not for MAO-A inhibition via interaction primarily driven by hydrophobic contacts. In conclusion, TR2 and TR16 are therapeutic prospects for the management of multiple neurodegenerative diseases.

## 1. Introduction

Parkinson’s disease (PD) is a neurological illness that affects 6.1 million individuals throughout the world [1]. Parkinsonism is a syndrome characterized by rigidity, bradykinesia, rest tremor, and postural instability [1,2,3]. PD is defined pathologically by the depletion of dopamine neurons in nigra pars compacta, which is followed by Lewy bodies, which are cytoplasmic inclusions within the midbrain comprising insoluble alpha-synuclein clusters. Nevertheless, PD is distinguished by more common pathology in different areas of the brain, which encompasses nondopaminergic neurons. [2]. The symptoms of disease can be treated with drugs that increase the level of dopamine, though they are not so effective.

Monoamine oxidases (MAO-A and MAO-B) are key enzymes that deaminate biogenic amines in the tissues of both peripheral and brain regions, controlling neurotransmitter levels including dopamine, norepinephrine, and epinephrine [4,5]. Selective MAO-A inhibitors have been shown to be useful in the management of depression [6]. Selective MAO-B inhibitors limit dopamine degradation in the central nervous system (CNS), blocking dopamine reduction. MAO-B inhibitors not only impede dopamine degradation, but also diminish the generation of neurotoxic byproducts of the MAO enzymatic reaction, such as hydrogen peroxide and aldehyde. L-Dopa has always remained the cornerstone of Parkinson’s therapy and continues to be the most efficacious suggestive therapeutic medication. L-Dopa is often used with selective MAO-B inhibitors. As a corollary, adopting MAO-B inhibitors in the initial phases of the disease may postpone the emergence of significant symptoms and the necessity for L-dopa. [7].

MAO inhibitors have various scaffolds with different structural frameworks such as anilide, benzothiazinone, chalcone, chromone, coumarin, enamide, hydrazone, indolalkylamine, pyrazoline, oxazolidinones, and propargylamine [8,9,10,11,12,13,14]. Pyridazinone is a six-membered cyclic hydrazine non-aromatic heterocyclic ring. The ring features in two conterminous nitrogen atoms, with one endocyclic double bond and one carbonyl functional moiety bearing from the unit. Diverse pharmacologic activity investigations on molecules with the 3(2*H*)-pyridazinone framework have been undertaken. Multiple bioactivities have really been documented for the compounds, including analgesic, anti-inflammatory, antihypertensive, cardiotonic, antiplatelet, anticholinesterase, anti-bacterial, antifungal, and antitumoral effects. [15,16]. In particular, pyridazinone derivatives are recognized as agents with noteworthy effects in the cardiovascular system attributed to their inhibition of platelet aggregation, antihypertensive activity, and cardiotonic qualities (Table 1).

In our previous reports, the synthesis of 3(2*H*)-pyridazinone candidates and the exploration of their MAO inhibitory properties were described. In the reports, new classes of pyridazinone core with substitution of benzalhydrazones, piperazine and morpholine analogues were designed and synthesized [17]. In addition, it was hypothesized that the addition of substituted benzalhydrazone to the second position of pyridazinone and the substitution of electron withdrawing groups in the benzalhydrazone ring caused MAO-B inhibitory activity to increase [18]. 

**Scheme 1:**
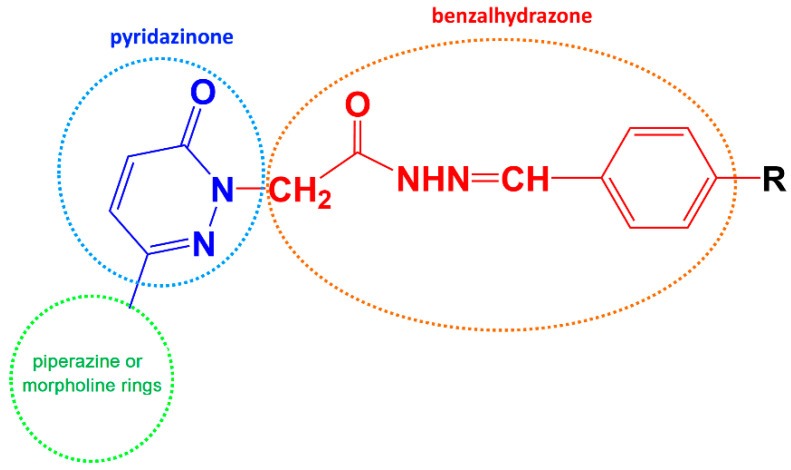
Rational design of the new hMAO inhibitors.

In particular, in this study, we aimed to synthesize, characterize, and determine the MAO-B inhibition activity of pyridazinone derivatives according to the hypothesis as shown below (Scheme 1).

## 2. Results and Discussion

### 2.1. General Chemistry of Synthetic Scheme

**Scheme 2:**
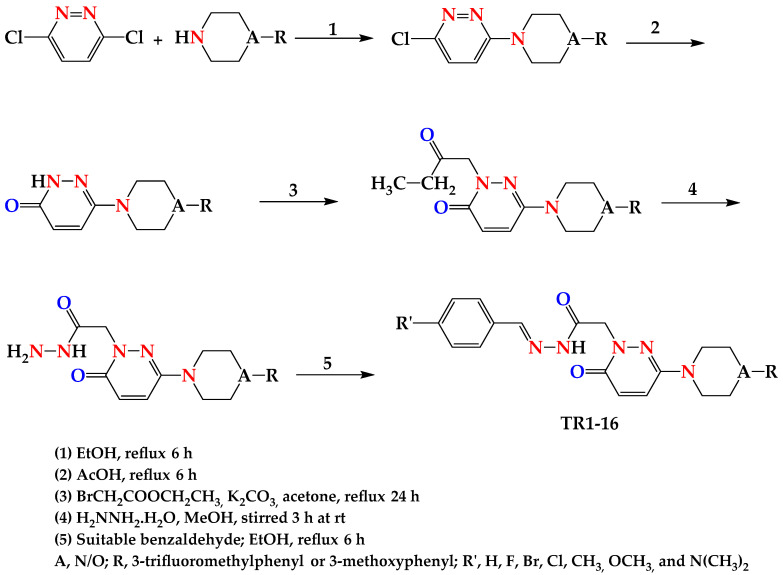
Synthesis of the compounds **TR1**-**16**.

The targeted pyridazinone compounds (**TR1**-**16**) were synthesized in accordance with the literature, as shown in Scheme 2.

The nucleophilic substitution reaction of 3,6-dichloropyridazine with phenylpiperazine or morpholine derivatives in ethanol was carried out to produce 3-chloro-6-substituted pyridazine. The hydrolysis of 3-chloro-6-(4-(3,4-dichlorophenyl)piperazine-1-yl)pyridazine by heating in glacial acetic acid afforded 6-(4-(3-Methoxy/3-trifluoromethylphenyl)piperazine-1-yl/morpholino)-3(*2H*)-pyridazinone [19]. Subsequently, the reaction of 6-(4-(3-methoxy/3-tifluoromethylphenyl)piperazine-1-yl/morpholino)-3(*2H*)-pyridazinone with ethyl bromoacetate in the presence of K_2_CO_3_ in acetone replaced the hydrogen atom of tertiary nitrogen atom in the pyridazinone ring and formed ethyl 6-(4-(3-methoxy/3-trifluoromethylphenyl)piperazine-1-yl/morpholino)-3(*2H*)-pyridazinone-2-ylacetate. The introduction of hydrazine hydrate (99%) can remove the ethyl group from the former intermediate and generate corresponding acid hydrazides [20]. Finally, nucleophilic addition reaction with various substituted aromatic aldehydes with acid hydrazides resulted in the formation of final targeted candidates (**TR1-TR16**). In this study, all of the title compounds were published for the first time. The compounds’ reaction yields vary from around 57% to 92%. The maximum yield (91.67%) was achieved with compound **TR8**, while the lowest yield was achieved with compound TR3 (57.10%). All structures were validated using ^1^H-NMR, ^13^C-NMR and mass spectral data. Table 2 lists the molecular structures and physical characterization of **TR1-TR16**.

Piperazine signals in compounds **TR1**–**13** were observed approximately between 3.2 and 3.5 ppm in ^1^H-NMR. Morpholine signals in compounds **TR14**–**16** were shifted between 3.2 and 3.68 ppm due to the oxygen atom in the ring (-OCH_2_-). Unlike other compounds **TR7**–**16**, compounds **TR1**–**6** with -OCH_3_ at the R^1^ position had a singlet signal at 3.80 ppm in ^1^H-NMR and a signal at 55 ppm in ^13^C-NMR. Compounds **TR5** and **TR11** with -CH_3_ at the R_2_ position were confirmed by the signal at 2.39 ppm in ^1^H-NMR and the signal at 21 ppm in ^13^C-NMR. **TR12** with 4-N(CH_3_)_2_ at the R_2_ position was confirmed by the signal at 3.04 ppm (6H, integral) in ^1^H-NMR.

### 2.2. Biochemistry

#### 2.2.1. MAO Inhibition Studies

At 10 μM, all compounds evaluated exhibited higher than 50% residual activity for MAO-A; however, only six compounds showed lower than 50% residual activity for MAO-B (Table 3). **TR16** had the strongest inhibitory action against MAO-B, with an IC_50_ value of 0.17 μM, followed by **TR2** (IC_50_ = 0.27 μM) (Table 3). The substituent -Cl atom at the *para* position of **TR16** showed 235.3- and 2.5-times higher MAO-B inhibitory activity over the basic structure **TR14** (IC_50_ = >40 μM) and -F atom at the *para* position of **TR15** (IC_50_ = 0.43 μM), respectively. On the other hand, the -Cl atom at the *para* position of **TR2** increased MAO-B inhibitory potency 148.1-fold (IC_50_ = 0.27 μM) compared to the basic structure **TR1** (IC_50_ = >40 μM). When TR2 and TR16 were compared, TR16 had a 1.59-fold higher IC_50_ for MAO-B than **TR2**. Both **TR2** and **TR16** showed selective inhibition for MAO-B with selectivity index (SI) values of >235.29 and 84.96, respectively (Table 3). The -OCH_3_ substituted derivatives at the R_1_ position (**TR1**-**6**) showed higher MAO-B inhibitory activity than the -CF_3_ substituted derivatives at the same position (**TR7**-**13**), except for the derivatives with -F substituent at the *para* position of R_2_, i.e., **TR3** and **TR9**. However, substitutions of –OCH_3_ and -CF_3_ at the R_1_ position did not show a significant difference in MAO-A inhibitory activity.

#### 2.2.2. Kinetic Study

**Figure 1:**
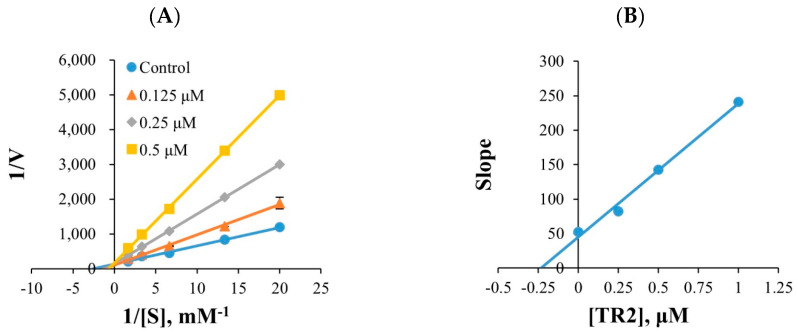

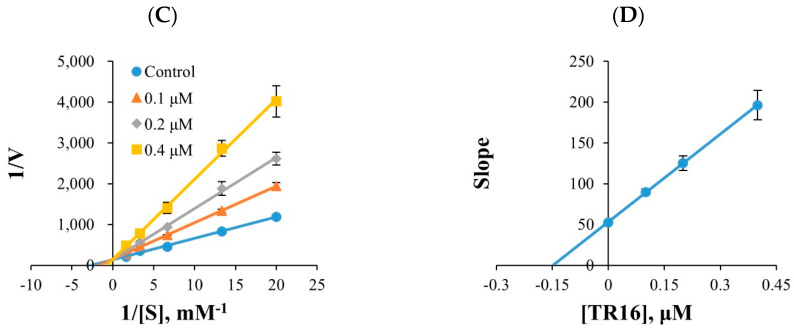
Lineweaver–Burk plots for MAO-B inhibition by **TR2** and **TR16** (**A**,**C**), and their respective secondary plots (**B**,**D**) of slopes versus inhibitor concentrations.

Five different substrate concentrations were used in the kinetics. **TR2** and **TR16** met a point on the y-axis in the Lineweaver–Burk plots (Figure 1A,C), and their secondary plots exhibited K_i_ values of 0.230 ± 0.004 and 0.149 ± 0.016 µM, respectively, in the kinetic investigations for MAO-B. (Figure 1B,D). **TR2** and **TR16** are competitive inhibitors that compete with the substrate and bind to the MAO-B active site.

#### 2.2.3. Reversibility Studies

**Figure 2:**
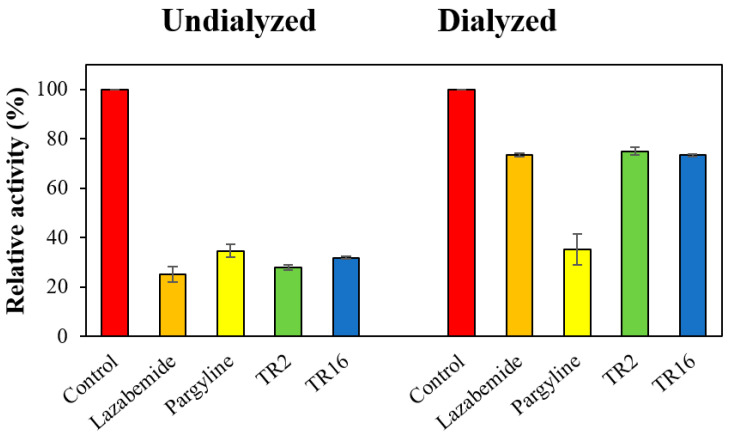
Recoveries of MAO-B inhibitions by **TR2** and **TR16** using dialysis experiments.

TR2 and TR16 concentrations in the reversibility studies were 0.54 and 0.34 μM, respectively, whereas the respective concentrations for the references, the reversible inhibitor lazabemide and the irreversible inhibitor pargyline, were 0.22 and 0.28 µM, respectively. The relative activity of undialyzed (AU) as well as dialyzed (AD) samples were compared to establish reversibility profiles. **TR2** and **TR16** inhibitions of MAO-B were restored from 28.0 % (AU) to 75.0 % (AD), and from 31.8% to 73.4%, respectively (Figure 2). When compared to the reference inhibitor, the compounds recovered to the levels comparable to lazabemide, the reference reversible inhibitor against MAO-B (varying between 25.0% and 73.4%), but were distinct from pargyline, a reference irreversible inhibitor against MAO-B. (i.e., varying between 34.5% and 35.2%). These data revealed that TR2 and TR16 were reversible inhibitors of MAO-B.

### 2.3. Parallel Artificial Membrane Permeability Assay (PAMPA)

The blood–brain barrier (BBB) permeation of the lead pyridaziones molecules (**TR2** and **TR16**) was confirmed by PAMPA [21]. The compounds showed good CNS permeabilities, with CNS+ positive standards such as progesterone and verapamil (Table 4).

### 2.4. Computational Studies

#### 2.4.1. Docking Studies

**Figure 3:**
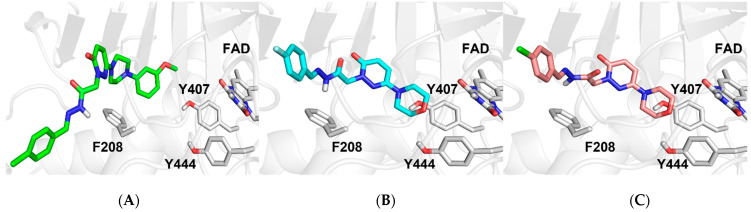
Zoomed in view at MAO-A binding site. Best poses of **TR2** (**A**), **TR15** (**B**), and **TR16** (**C**) were reported as green, cyan, and magenta sticks, respectively. For completeness, docking scores returned from simulations were equal to −7.99, −8.29, and −8.16 kcal/mol for **TR2**, **TR15**, and **TR16**, respectively.

**Figure 4:**
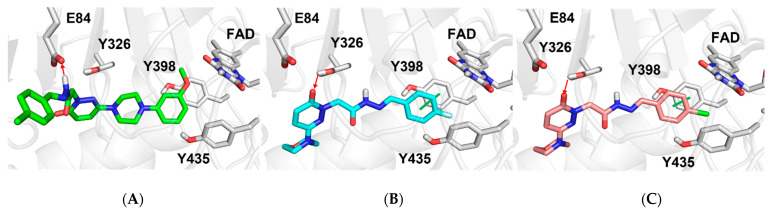
Zoomed in view at MAO-B binding site. Best poses of **TR2** (**A**), **TR15** (**B**), and **TR16** (**C**) were reported as green, cyan, and magenta sticks, respectively. Red arrows and green lines indicate hydrogen bonds and π–π contacts, respectively. For completeness, docking scores returned from simulations were equal to −10.051, −9.452 and −8.558 kcal/mol for **TR2**, **TR15** and **TR16**, respectively.

Molecular modeling simulations were performed to give a sound indication of molecular interactions responsible for MAO-A and MAO-B inhibitions. Figure 3 and Figure 4 show the best poses returned from docking simulations. For completeness, the 2D interaction schemes were enclosed as Supporting Information in Figures S49 and S50.

Compounds **TR2, TR15,** and **TR16** interact within the MAO-A binding site through hydrophobic contacts. Morpholine ring of **TR15** and **TR16** faces the aromatic rings of FAD, Y407, and Y444, a binding room not engaged by the *meta*-methoxy ring of **TR2**, due to its bulky structure. 

**Figure 5:**
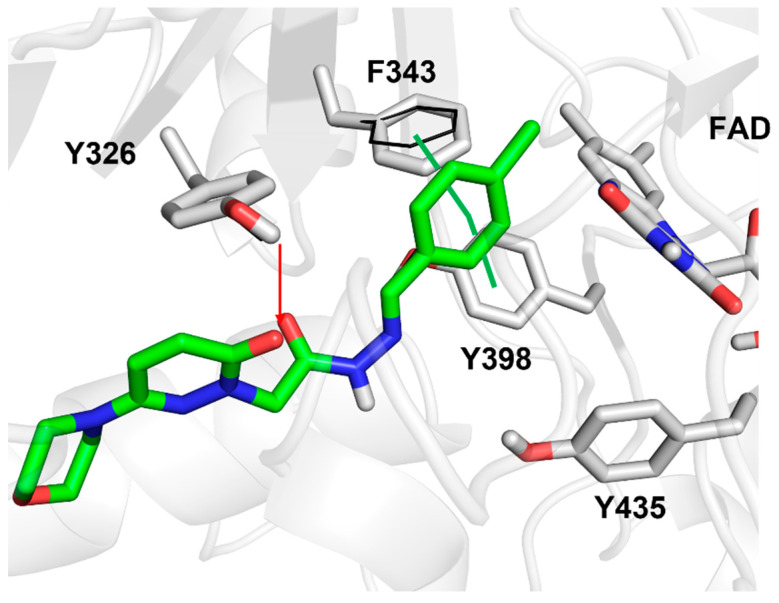
Best docked pose of **TR16** resulted from the induced-fit docking protocol. Green lines and red arrows depict π–π contacts and hydrogen bonds, respectively. Black lines show the original conformation of MAO-B sidechains. For completeness, docking score was equal to −11.544 kcal/mol.

As shown in Figure 4, compound **TR2** reaches the aromatic cage formed by FAD, Y398, and Y435 through its meta-methoxy ring and establishes a hydrogen bond with the side chain of E84, a MAO-B selective residue (changed to V93 in MAO-A). Moreover, compounds **TR15** and **TR16** engage the same interactions with Y398 through π–π contacts and with the phenol group of MAO-B selective residue Y326 by forming a hydrogen bond with the carbonyl groups of the pyridazinone rings. Overall, molecular docking analyses suggested that compounds were not very effective at engaging the MAO-A binding pocket, due to the interaction mainly driven by hydrophobic contacts. Finally, the induced-fit docking protocol was used in order to deepen the study concerning the interactions occurring between **TR16** and MAO-B (Figure 5). 

As reported in Figure 5, the binding mode of **TR16** was consistent with previous standard docking simulation. In detail, the interactions with selective residue Y326 and with Y398 were detected, and an additional aromatic residue, F343, was recruited to engage a new π–π contact, with a little shift from its initial position. 

#### 2.4.2. Bioavailability Prediction

**Figure 6:**
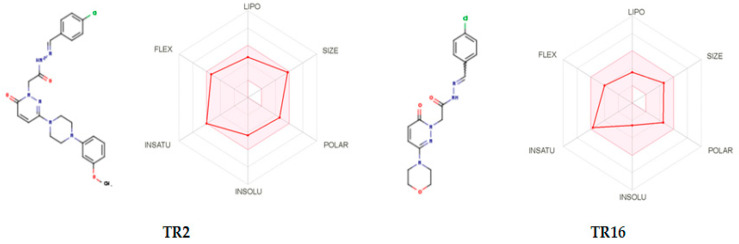
The bioavailability radar of **TR2** and **TR16**.

The bioavailability of lead molecules was predicted by SwissADME online platform. The bioavailability radar panel assesses drug candidates based on physicochemical parameters such as lipophilicity, flexibility, polarity insolubility, size, and saturation [22]. Interestingly, both **TR2** and **TR16** molecules had been reported to be in the pink region (optimal ranges) and to have drug-like properties (Figure 6).

## 3. Materials and Methods

### 3.1. Chemistry

The synthetic procedures of the titled compounds are provided in the supplementary information.

### 3.2. Biological Analysis

#### 3.2.1. Enzyme Assays

Recombinant MAO-A and MAO-B were used in MAO inhibitory activity assays using kynuramine (0.06 mM) and benzylamine (0.3 mM) as substrates, respectively [23]. The MAO-A and MAO-B reactions were carried out at 25 °C and monitored for 30 min at 316 and 250 nm, respectively, in 0.5 mL mixture of 50 mM sodium phosphate (pH 7.2). Clorgyline and toloxatone were the drug references used for MAO-A, and pargyline and lazabemide were used for MAO-B as reference compounds. The IC_50_ values were determined by measuring the residual activity at different concentrations of the compounds and by using GraphPad Prism software 5.0. The K_m_ value of benzylamine against MAO-B was measured to be 0.40 mM [24]. On the other hand, multitarget analysis, ChE (AChE, BChE) inhibitory activity assays were carried out at 25 °C and monitored for 15 min at 412 nm in a 0.5 mL mixture of 100 mM sodium phosphate (pH 7.5). BACE1 inhibitory activities were tested by the BACE1 activity detection kit. The assay method was described previously [25]. Recombinant human MAO-A and MAO-B, AChE from *Electrophorus electricus*, BChE from equine serum, BACE1, and their substrates and reference inhibitors were purchased from Sigma-Aldrich (St. Louis, MO, USA).

#### 3.2.2. Inhibition Profile of MAOs and Kinetic Studies

**TR1** and **TR16** MAO inhibitory actions were evaluated at 10 μM for primary screening. For the compounds with <80% residual activities, their IC_50_ values were calculated. **TR2** and **TR16** kinetics with MAO-B were investigated at five different substrate concentrations (0.0375–0.6 μM). Lineweaver–Burk plots were used to examine the inhibition patterns, and secondary plots were used to calculate the K_i_ values with three inhibitor dosages [26,27,28].

#### 3.2.3. Inhibition Reversibility of TR2 and TR16

After preincubation with the enzyme for 30 min at ~2 × IC_50_ (i.e., 0.54 and 0.34 μM, respectively), the reversibility of **TR2** or **TR16** against MAO-B inhibition was evaluated using a dialysis approach and employing a dialysis kit, as previously described [29,30]. Lazabemide (a reversible MAO-B inhibitor) and pargyline (an irreversible MAO-B inhibitor) were preincubated at 0.22 and 0.28 μM, respectively, as reference compounds. To determine reversibility patterns, the activities of dialyzed (AD) and undialyzed (AU) specimens were compared.

### 3.3. BBB Study by PAMPA Method

Using the PAMPA method, blood–brain barrier permeation abilities of lead compounds **TR2** and **TR16** were analyzed [21].

### 3.4. Docking Studies

X-ray crystallographic structures of MAO-A and MAO-B were taken from the Protein Data Bank by finding records 2Z5X and 2V5Z, respectively [31,32]. With the assistance of the protein preparation wizard tool, both protein structures were evaluated for the ionization states of acid/basic side chains at physiological pH, to remove nonfunctional water and to carry out energy minimization steps [33,34]. Ligands to be docked were prepared by using the Ligprep tool to sample allowed tautomers and ionization states for all the possible conformers. Standard docking protocol (SP) was carried out using GLIDE by utilizing the OPLS3 force field and setting 50,000 poses per ligand for the first phase and 4000 poses per ligand for energy minimization [35,36,37]. Best poses yielded Root Mean Square Deviation (RMSD) values of 0.39 Å and 0.76 Å for MAO-A and MAO-B cognate ligands, respectively, which was satisfactory. Eventually, the induced-fit docking protocol was employed in order to better understand the interactions between compound **TR16** and MAO-B. This protocol allows the conformational changes in residues involved in the ligand binding to be considered, thus enhancing the reliability and the accuracy of computational simulations [38]

### 3.5. Bioavailiability Predicition

The bioavailability prediction of lead molecules **TR2** and **TR16** was assessed at http://www.swisstargetprediction.ch [39] (accessed on 1 March 2022).

## 4. Conclusions

The current work evaluated the MAO-A and MAO-B inhibitory characteristics of a series of sixteen pyridazinone derivatives that were synthesized. The majority of the chemicals inhibited MAO-B more effectively than MAO-A. The lead compounds TR2 and TR16 have been shown to be competitive and reversible MAO-B inhibitors in kinetic and reversibility experiments. The PAMPA test revealed that the lead candidates have a high level of CNS permeability. According to docking studies, the strong inhibitory efficiency of compounds TR2 and TR16 is due to the possibility of interaction with important and selective residues of E84 or Y326 in MAO-B. These findings are consistent with the experimental data. The compounds’ pharmacological similarity was also shown by the favorable bioavailability radar.

## Figures and Tables

**Table 1:** Some therapeutic drugs with pyridazinone core.

Structure	Compound	Activity
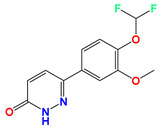	Zardaverine	Cardiotonic
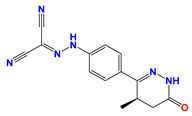	Levosimendan	Vasodilator
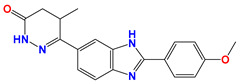	Pimobendan	Vasodilator
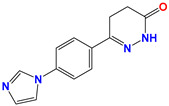	Imazodan	Cardiotonic
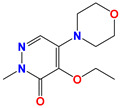	Emorfazone	Analgesic
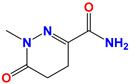	Medazomide	Antitussif

**Table 2:** Chemical-physical data of titled compounds.

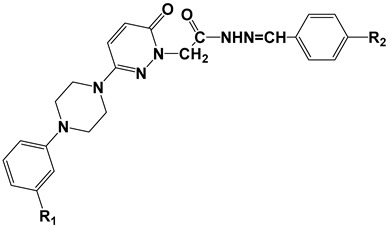					
**Entry**	**R_1_**	**R_2_**	**Yield (%)**	**M.P. (°C)**	**Molecular Formula**
**TR1**	OCH_3_	H	79.23	232–235	C_24_H_26_N_6_O_3_
**TR2**	OCH_3_	4-Cl	82.49	205	C_24_H_25_ClN_6_O_3_
**TR3**	OCH_3_	4-F	57.10	172	C_24_H_25_FN_6_O_3_
**TR4**	OCH_3_	4-OCH_3_	67.08	154	C_25_H_28_N_6_O_4_
**TR5**	OCH_3_	4-CH_3_	76.73	201–202	C_25_H_28_N_6_O_3_
**TR6**	OCH_3_	4-Br	82.54	214	C_24_H_25_BrN_6_O_3_
**TR7**	CF_3_	H	76.90	126	C_24_H_23_F_3_N_6_O_2_
**TR8**	CF_3_	4-Cl	91.67	242	C_24_H_22_ClF_3_N_6_O_2_
**TR9**	CF_3_	4-F	78.09	224	C_24_H_22_F_4_N_6_O_2_
**TR10**	CF_3_	4-OCH_3_	86.36	195	C_25_H_25_F_3_N_6_O_3_
**TR11**	CF_3_	4-CH_3_	83.43	142	C_25_H_25_F_3_N_6_O_2_
**TR12**	CF_3_	4-N(CH_3_)_2_	80.21	227	C_26_H_28_F_3_N_7_O_2_
**TR13**	CF_3_	4-Br	82.64	241	C_24_H_22_BrF_3_N_6_O_2_
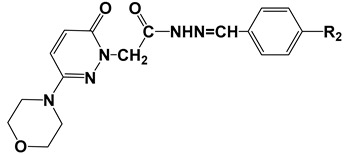					
**Entry**	**R_2_**		**Yield (%)**	**M.P. (°C)**	**Molecular Formula**
**TR14**	H		71.22	206	C_17_H_19_N_5_O_3_
**TR15**	4-F		61.17	119	C_17_H_18_FN_5_O_3_
**TR16**	4-Cl		80.73	152	C_17_H_18_ClN_5_O_3_

**Table 3:** Inhibition of MAO-A and MAO-B by the **TR** series ^a^.

Compounds	Residual Activity at 10 µM (%)		IC_50_ (µM)		SI ^b^
	MAO-A	MAO-B	MAO-A	MAO-B	
**TR1**	82.04 ± 1.20	80.54 ± 1.01	17.32 ± 1.20	>40	<0.43
**TR2**	65.85 ± 0.66	−4.37 ± 4.13	22.94 ± 0.24	0.27 ± 0.02	84.96
**TR3**	71.66 ± 0.71	20.11 ± 1.15	19.65 ± 0.30	2.11 ± 0.51	9.31
**TR4**	61.00 ± 3.54	88.64 ± 8.61	16.53 ± 1.66	>40	<0.41
**TR5**	72.83 ± 1.02	45.56 ± 2.56	18.19 ± 0.09	4.42 ± 0.23	4.12
**TR6**	51.73 ± 4.55	70.23 ± 3.80	12.33 ± 0.11	15.12 ± 0.18	0.81
**TR7**	55.40 ± 0.84	90.66 ± 6.64	14.43 ± 3.55	>40	<0.36
**TR8**	52.87 ± 0.82	69.09 ± 0.95	12.02 ± 1.75	20.00 ± 0.00	0.60
**TR9**	72.28 ± 0.40	46.51 ± 4.93	17.08 ± 0.21	1.00 ± 0.15	17.08
**TR10**	56.00 ± 2.26	91.86 ± 7.09	13.28 ± 1.81	>40	<0.33
**TR11**	55.65 ± 2.28	83.87 ± 0.56	18.54 ± 5.83	32.00 ± 1.21	0.57
**TR12**	52.55 ± 1.77	85.33 ± 1.59	14.72 ± 2.96	>40	<0.37
**TR13**	74.24 ± 6.43	100.32 ± 5.43	31.00 ± 4.33	>40	<0.78
**TR14**	97.47 ± 0.71	106.69 ± 2.01	>40	>40	1.00
**TR15**	85.59 ± 1.04	12.50 ± 3.54	>40	0.43 ± 0.05	>93.02
**TR16**	85.00 ± 3.08	13.06 ± 0.64	>40	0.17 ± 0.04	>235.29
Toloxatone			1.08 ± 0.03	-	
Lazabemide			-	0.11 ± 0.02	
Clorgyline			0.007 ± 0.001	-	
Pargyline			-	0.14 ± 0.01	

^a^ The results represent the averages of duplicate or triple studies, including standard errors. ^b^ SI values are presented for MAO-B compared with MAO-A. The IC_50_ concentration was determined to be 40 µM as the highest concentration.

**Table 4:** BBB permeation of standard and TR compounds by PAMPA.

Compounds	Bibliography Pe (×10^−6^ cm/s) ^a^	Experimental Pe (×10^−6^ cm/s)	Prediction
Dopamine	0.2	0.21 ± 0.01	CNS-
Lomefloxacin	1.1	1.13 ± 0.01	CNS-
Verapamil	16.0	15.35 ± 0.33	CNS+
Progesterone	9.3	9.02 ± 0.17	CNS+
**TR2**		9.33 ± 0.33	CNS+
**TR16**		10.62 ± 0.26	CNS+

CNS-: Pe (10^−6^ cm/s) <2, less BBB permeable; CNS+, Pe (10^−6^ cm/s) >4, good BBB permeable. ^a^ from [21].

## Data Availability

MDPI Research Data Policies” at https://www.mdpi.com/ethics (accessed on 25 February 2022).

## Supplementary Materials

The following supporting information can be downloaded at: https://www.mdpi.com/article/10.3390/molecules27123801/s1, Experimental procedures for the synthesis of the titled compounds. Figures S1–S48; ^1^H-NMR, ^13^C-NMR, and HRMS spectra of the compounds **TR1**~**16**. Figures S49 and S50; 2D-schemes of **TR2**, **TR15**, and **TR16** interactions to MAO-B.

## Sample Availability

Samples of the compounds **TR1** to **TR16** are available from the authors.
