# Supplementary material for: Development of a Novel Class of Pyridazinone Derivatives as Selective MAO-B Inhibitors
Source: Molecules. 2022 Jun 13;27(12):3801. doi: 10.3390/molecules27123801 (PMC9230784; doi:10.3390/molecules27123801)
Supplement: Supplementary file 1 [file molecules-27-03801-s001.zip › molecules-1674654-supplementary.pdf]

molecules

MDPI

SUPPORTING INFORMATION

Development of A Novel Class of Pyridazinone Derivatives as Selective MAO-B Inhibitors

Mehmet Abdullah Alagöz [1,#], Jong Min Oh [2,#], Yaren Nur Zenni [1], Zeynep Özdemir [1], Mohamed A Abdelgawad [3], Ibrahim A. Naguib [4], Mohammed M. Ghoneim [5], Nicola Gambacorta [6], Orazio Nicolotti [6], Hoon Kim [2,*] and Bijo Mathew [7,*]

[1] Department of Pharmaceutical Chemistry, Inonu University, Faculty of Pharmacy, Malatya 44280, Turkey; mehmet.alagoz@inonu.edu.tr (M.A.A.); yarennur.zenni@ inonu.edu.tr (Y.N.Z.); zeynep.bulut@inonu.edu.tr (Z. Ö.)
[2] Department of Pharmacy, and Research Institute of Life Pharmaceutical Sciences, Sunchon National University, Suncheon 57922, Republic of Korea; ddazzo005@naver.com (J.M.O.)
[3] Department of Pharmaceutical Chemistry, College of Pharmacy, Jouf University, Sakaka, Al Jouf 72341, Saudi Arabia; mhmdgwd@ju.edu.sa (M.A.A.)
[4] Department of Pharmaceutical Chemistry, College of Pharmacy, Taif University, P.O. Box 11099, Taif 21944, Saudi Arabia; i.abdelaal@tu.edu.sa (I.A.N.)
[5] Department of Pharmacy Practice, Faculty of Pharmacy, AlMaarefa University, Ad Diriyah 13713, Saudi Arabia; mghoneim@mcst.edu.sa (M.M.G.)
[6] Dipartimento di Farmacia—Scienze del Farmaco, Università degli Studi di Bari "Aldo Moro", Via E. Orabona, 4, I-70125 Bari, Italy; nicola.gambacorta1@uniba.it (N.G.); orazio.nicolotti@uniba.it (O.N.)
[7] Department of Pharmaceutical Chemistry, Amrita School of Pharmacy, Amrita Vishwa Vidyapeetham, AIMS Health Sciences Campus, Kochi-682 041, India.
* Correspondence: bijomathew@aims.amrita.edu or bijovilaventgu@gmail.com (B.M.); hoon@sunchon.ac.kr (H.K.)
[#] These authors contributed equally to this work.

Contents

1. Experimental procedures for the synthesis of the titled compounds
2. ^{1}H-NMR, ^{13}C-NMR, and HRMS spectra of the compounds **TR1~16** Figures S1–S48
3. 2D-schemes of **TR2, TR15,** and **TR16** interactions to MAO-B Figures S49 and S50

1.1. Experimental procedure for synthesis

For synthesis 3-chloro-6-[4-(3-trifluoromethyl/3-methoxyphenyl)piperazine-1-yl] pyridazine/3-chloro-6-(morpholine) pyridazine, 0.01 mol of 3,6-dichloropyridazine and 0.01 mol of (3-trifluoromethyl/3-methoxyphenyl)piperazine/morpholine were mixed in 15 mL of ethanol under reflux with heating for 6 h according to the literature procedure [19]. The reaction liquid was emptied into freezing water; the precipitate produced by filtration was purified by crystallization from ethanol. The melting points of non-original compounds were consistent with previous research. To manufacture 6-[4-(3-trifluoromethyl/3-methoxyphenyl)piperazine-1-yl]/6-morpholine-3(2H)-pyridazinone, 0.05 mol of 3-chloro-6-[4-[4-(3-trifluoromethyl/3-methoxyphenyl)piperazine-1-yl]pyridazine/3-chloro-6-(morpholine) pyridazine was refluxed for 6 h in 30 mL glacial acetic acid. Acetic acid was removed at reduced pressure; the residue was dissolved in water and extracted with chloroform. The organic phase was evaporated under decreased pressure after being dried on sodium sulphate. Recrystallization from ethanol was used to purify the residue [19, 20]. For synthesis ethyl 6-[4-(3-trifluoromethyl/3-

methoxyphenyl)piperazine-1-yl]/6-morpholine-3(2H)-pyridazinone-2-ylacetate, 0.01 mol of 6-(4-(3-methoxyphenyl/4-trifluoromethyl)piperazin-1-yl)pyridazin-3(2*H*)-one, 0.02 mol of ethyl bromoacetate, and 0.02 mol of potassium carbonate were mixed and refluxed overnight in acetone (40 mL). After cooling, the organic salts were filtered out, the solvent was evaporated, and the residue was purified by recrystallization with n-hexane to yield the esters. Next, 0.01 mol 6-[4-(3-trifluoromethyl/3-methoxyphenyl)piperazine-1-yl]/6-morpholine-3(2H)-pyridazinone-2-ylacetate, and hydrazine hydrate (99 percent, 3 mL) were dissolved in 25 mL methanol and stirred for 3h at room temperature. The resulting precipitate was filtered, washed with water, dried, and recrystallized from ethanol [18].

1.2. General procedure for synthesis of the title compounds (**TR1-16**)

A solution of 0.01 mol 6-[4-(3-trifluoromethyl/3-methoxyphenyl)piperazine-1-yl]/6-morpholine-3(2H)-pyridazinone-2-ylacetohydrazide and 0.01 mol substituted/nonsubstituted benzaldehyde was agitated and refluxed for 6 h in ethanol (15 mL). The mixture was placed into ice water at the end of the process. From methanol:water, the precipitate was filtered, dried, and crystallized [17-19]. All of the spectral data from the five novel compounds were consistent with the given structures, in supplementary data.

Figure S1. ^{1}H-NMR spectra of compound **TR1**

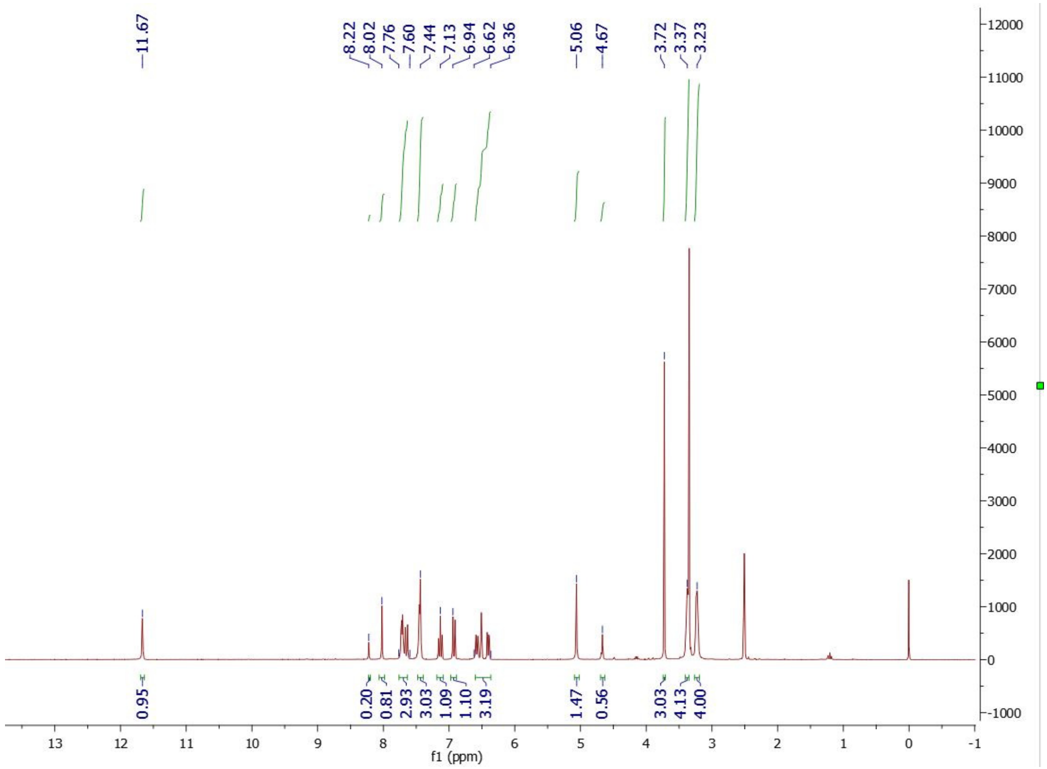

2

Figure S2. ^{13}C-NMR spectra of compound **TR1**

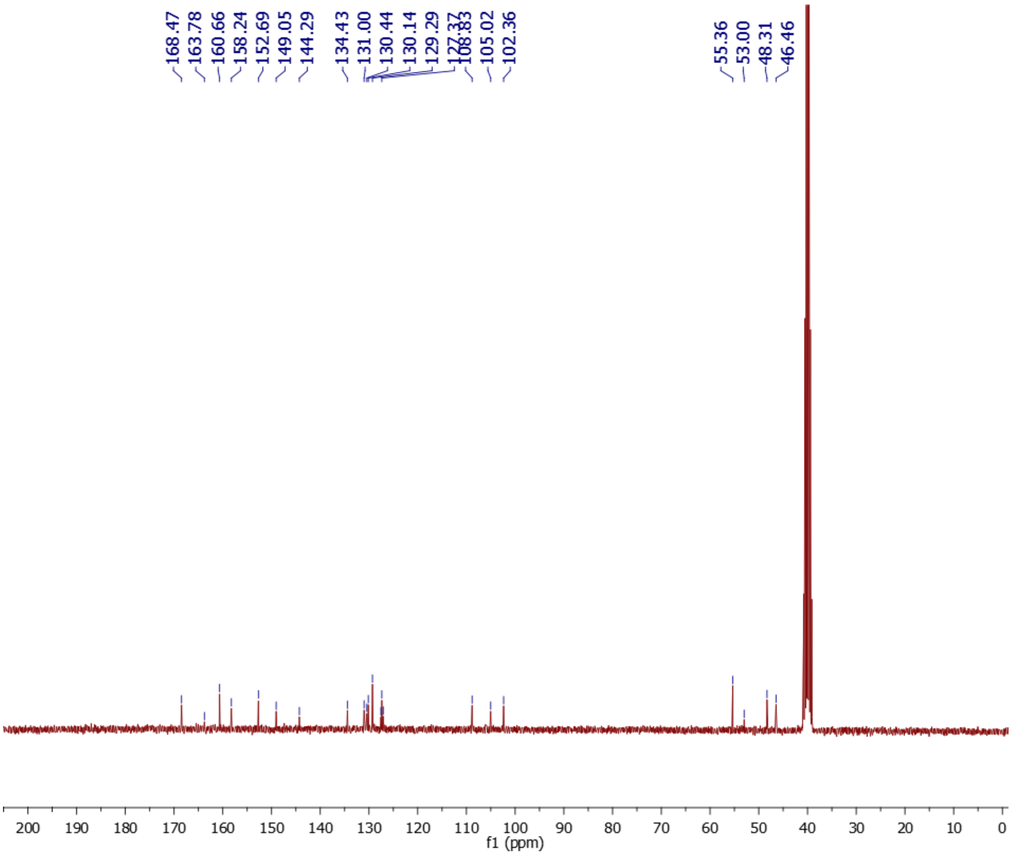

Figure S3. HRMS spectra of compound **TR1**

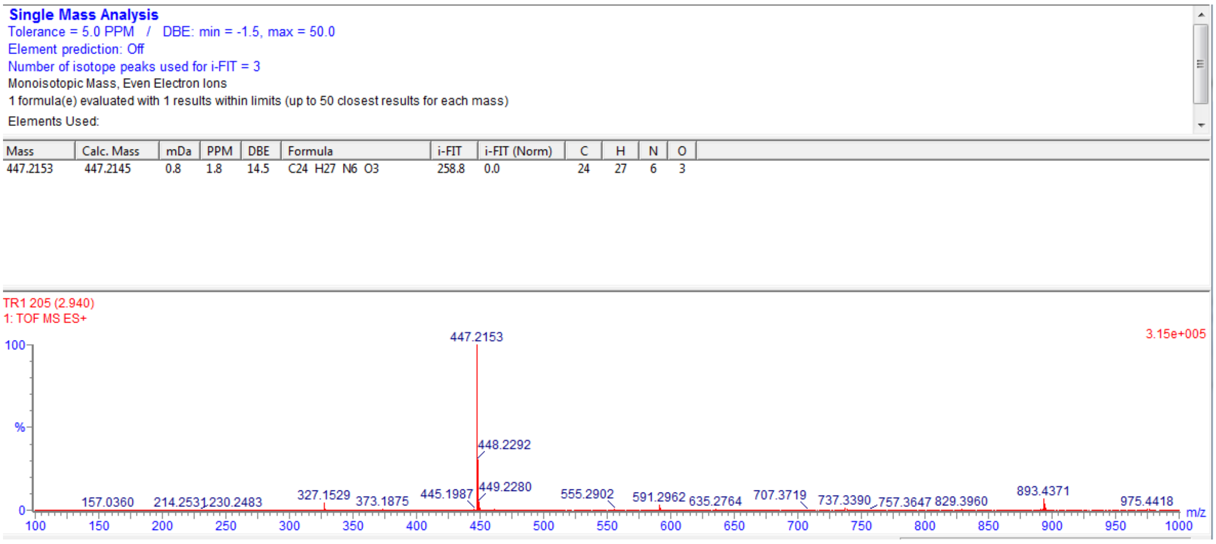

N'-benzylidene-2-(3-(4-(3-methoxyphenyl)piperazin-1-yl)-6-oxopyridazin-1(6H)-yl)acetohydrazide (**TR1**)

^{1}H-NMR (DMSO-d_6, 300 MHz): δ 3.23 (4H; t; CH_2N; b+b'), 3.37 (4H; t; CH_2N; a+a'), 3.72 (3H; s; OCH_3), 5.06 (2H; s; CH_2CO), 6.39-7.73 (11H; m; phenyl protons, pyridazinone $H^{4,5}$), 8.02 (1H; s; -N=CH-) and 11.67 (1H; s; -NH-N).

^{13}C-NMR (DMSO-d$_6$, 300 MHz), δ 46.46 (2C, CH$_2$-N; b+b'), 48.31 (2C, CH$_2$-N; a+a'), 53.00 (1C; -N-C̲H$_2$-C=O), 55.36 (1C, OCH$_3$), 102.36 (1C; =CH), 105.02 (1C; phenyl C^4), 108.83 (2C; phenyl C^{3,5}), 127.37 (2C; phenyl C^{2,6}), 129.29 (1C; phenyl C^1), 130.14 (1C; 3-methoxyphenyl C^5), 130.44 (1C; pyridazinone C^4), 131.00 (1C; 3-methoxyphenyl C^6), 134.43 (1C; 3-methoxyphenyl C^1), 144.29 (1C; 3-methoxyphenyl C^4), 149.05 (1C; 3-methoxyphenyl C^2), 152.69 (1C; pyridazinone C^5), 158.24 (1C; pyridazinone C^6), 160.66 (1C; 3-methoxyphenyl C^3), 163.78 (1C; CH$_2$-N-C̲=O), 168.47 (1C; pyridazinone C^3);

$C_{24}H_{27}N_6O_3$ MS (ESI+) calculated: 447.2145, Found: m/e 447.2153(M$^+$; 100.0%).

Figure S4. ^{1}H-NMR spectra of compound **TR2**

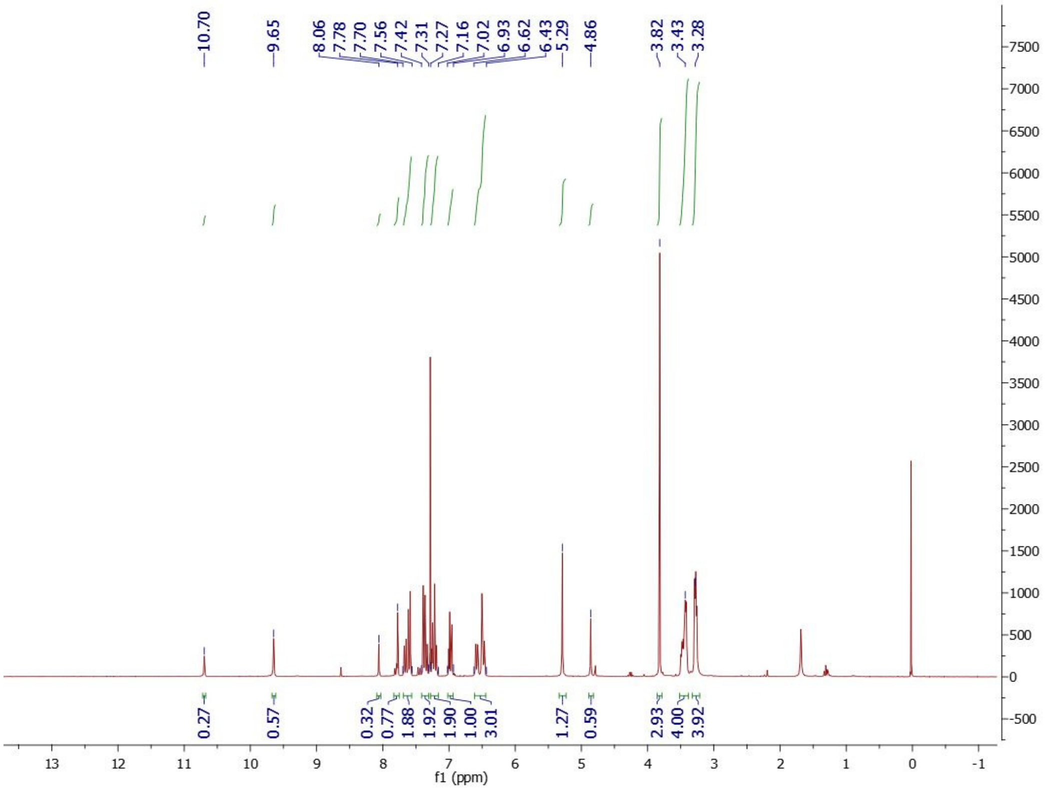

Figure S5. ^{13}C-NMR spectra of compound **TR2**

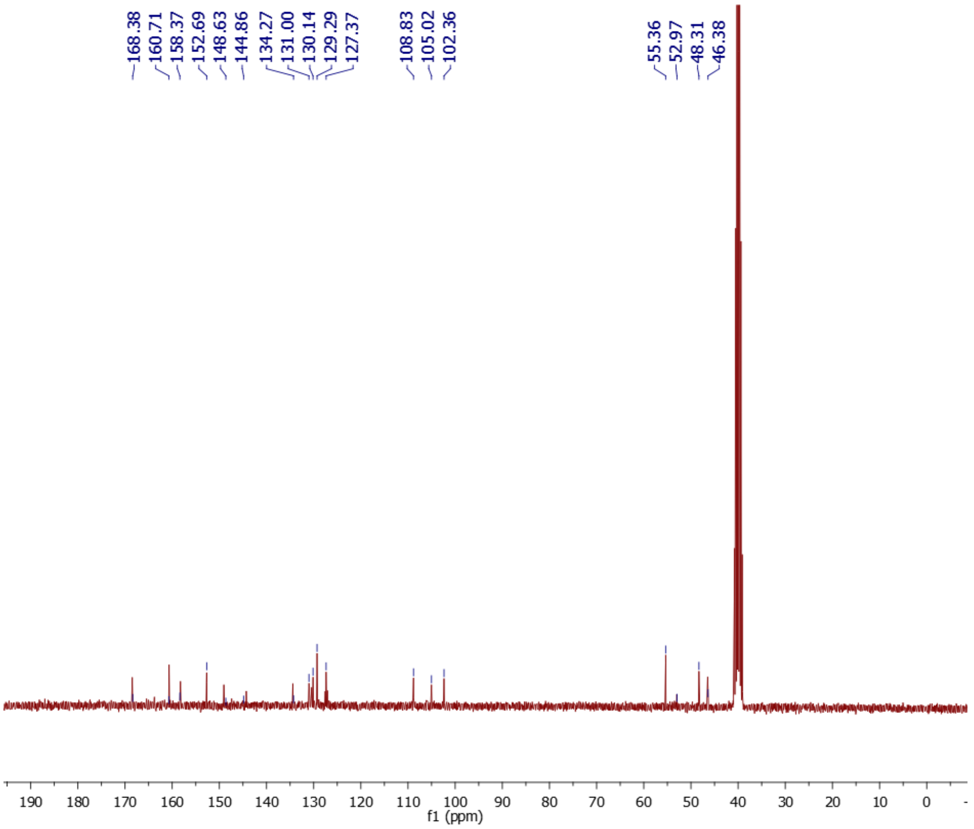

Figure S6. HRMS spectra of compound **TR2**

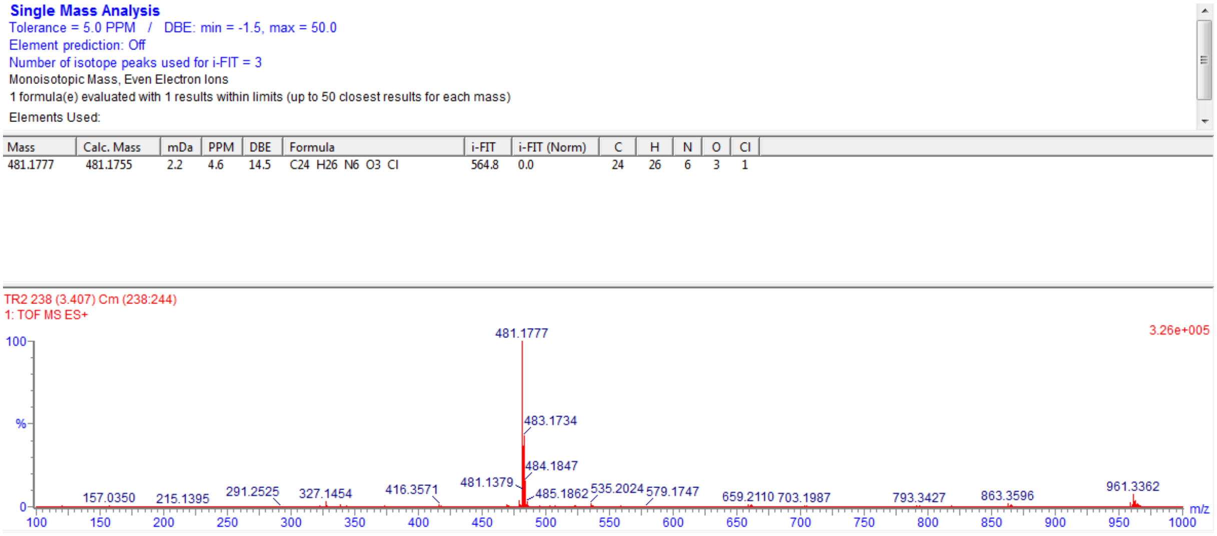

N'-(4-chlorobenzylidene)-2-(3-(4-(3-methoxyphenyl)piperazin-1-yl)-6-oxopyridazin-1(6H)-yl)acetohydrazide (**TR2**)

^{1}H-NMR (DMSO-d_6, 300 MHz): δ 3.27 (4H; t; CH_2N; b+b′), 3.43 (4H; t; CH_2N; a+a′), 3.82 (3H; s; OCH_3), 5.29 (2H; s; CH_2CO), 6.46-7.68 (10H; m; phenyl protons, pyridazinone $H^{4,5}$), 8.06 (1H; s; -N=CH-) and 9.65 (1H; s; -NH-N).

^{13}C-NMR (DMSO-d_6, 300 MHz), δ 46.38 (2C, CH2-N; b+b′), 48.31 (2C, CH2-N; a+a′), 52.97 (1C; -N-C̲H2-C=O), 55.36 (1C, OCH_3), 102.36 (1C; =CH), 105.02 (4C; 4-chlorophenyl $C^{2,3,5,6}$), 108.83 (1C; 4-chlorophenyl C^1), 127.37 (1C; pyridazinone C^4), 129.29 (1C; 3-methoxyphenyl C^1), 130.14 (3C; 3-methoxyphenyl $C^{4,5,6}$), 131.00 (1C; 3-methoxyphenyl C^2), 134.27 (1C; pyridazinone C^5), 144.86 (1C; pyridazinone C^6), 148.63 (1C; 4-chlorophenyl C^4), 152.23 (1C; 3-methoxyphenyl C^3), 158.37 (1C; CH2-N-C=O), 168.38 (1C; pyridazinone C^3);

$C_{24}H_{26}ClN_6O_3$ MS (ESI+) calculated: 481.1755, Found: m/e 481.1777(M^+; 100.0%).

Figure S7. ^{1}H-NMR spectra of compound **TR3**

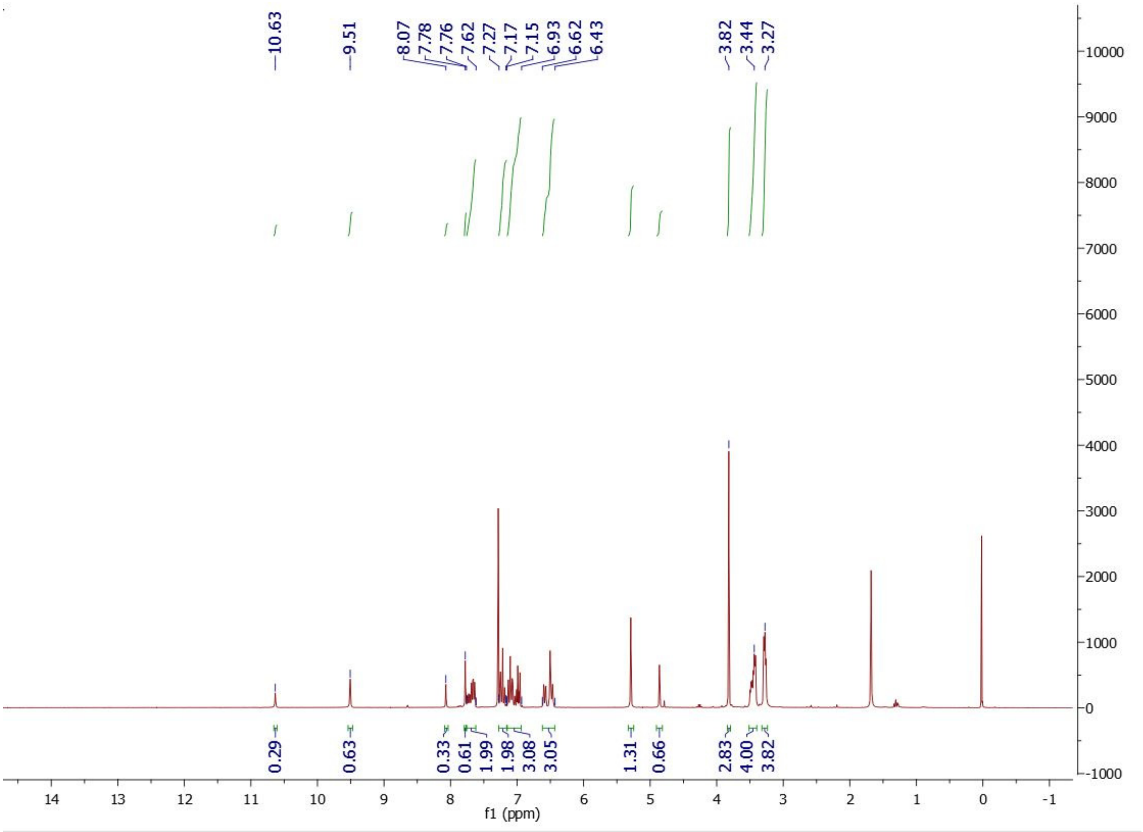

Figure S8. ^{13}C-NMR spectra of compound **TR3**

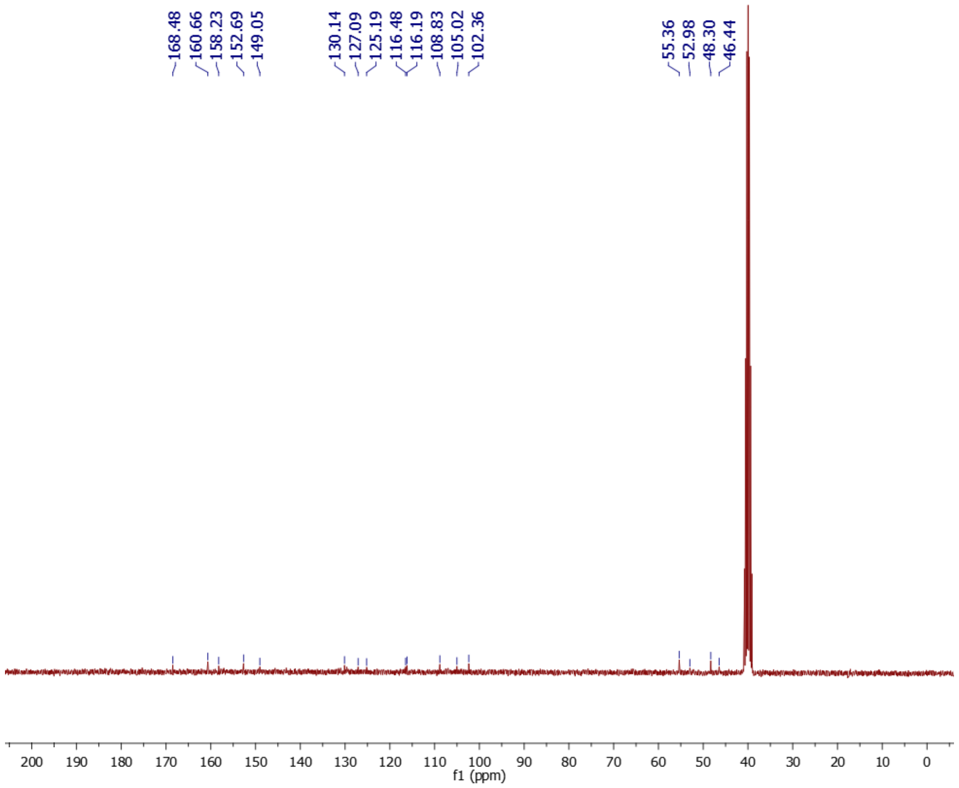

Figure S9. HRMS spectra of compound **TR3**

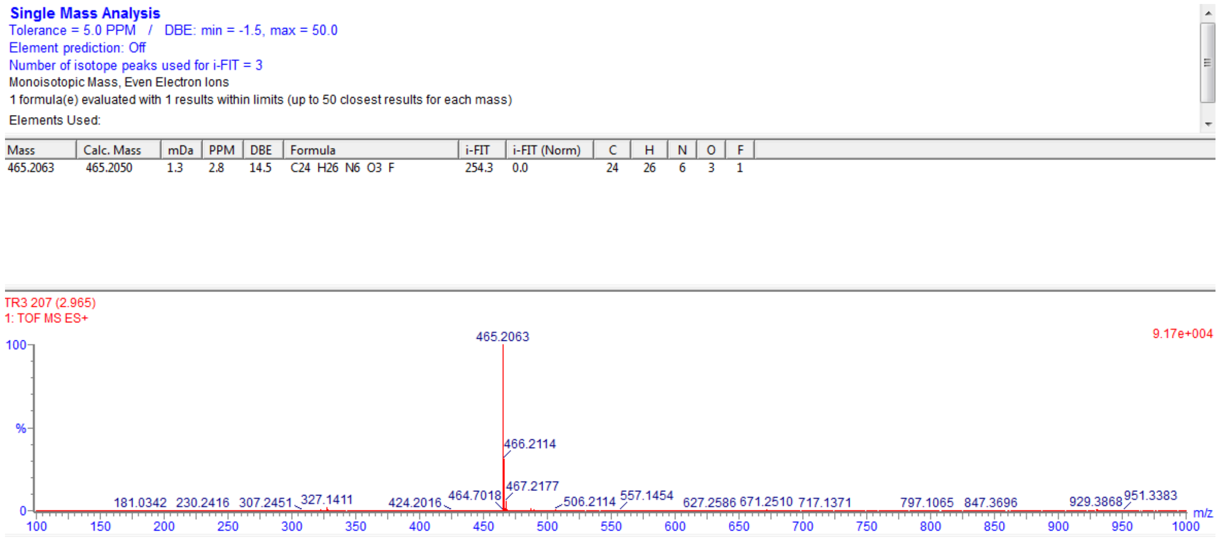

N'-(4-fluorobenzylidene)-2-(3-(4-(3-methoxyphenyl)piperazin-1-yl)-6-oxopyridazin-1(6H)-yl)acetohydrazide (**TR3**)

^{1}H-NMR (DMSO-d_6, 300 MHz): δ 3.27 (4H; t; CH_2N; b+b'), 3.45 (4H; t; CH_2N; a+a'), 3.82 (3H; s; OCH_3), 5.29 (2H; s; CH_2CO), 6.44-7.75 (10H; m; phenyl protons, pyridazinone $H^{4,5}$), 8.07 (1H; s; -N=CH-) and 9.51 (1H; s; -NH-N).

^{13}C-NMR (DMSO-d_6, 300 MHz), δ 46.44 (2C, CH2-N; b+b'), 48.30 (2C, CH2-N; a+a'), 52.98 (1C; -N-C̲H2-C=O), 55.36 (1C, OCH_3), 102.36 (1C; =CH), 105.02 (4C; 4-fluorophenyl

$C^{2,3,5,6}$), 108.83 (1C; 4-fluorophenyl C^{1}), 116.19 (1C; pyridazinone C^{4}), 116.48 (1C; 3-methoxyphenyl C^{1}), 125.19 (3C; 3-methoxyphenyl $C^{4,5,6}$), 127.09 (1C; 3-methoxyphenyl C^{2}), 130.14 (1C; pyridazinone C^{5}), 149.05 (1C; pyridazinone C^{6}), 152.69 (1C; 4-fluorophenyl C^{4}), 158.23 (1C; 3-methoxyphenyl C^{3}), 160.66 (1C; CH2-N-C=O), 168.48 (1C; pyridazinone C^{3});

$C_{24}H_{26}FN_6O_3$ MS (ESI+) calculated: 465.2050, Found: m/e 465.2063 (M^+; 100.0%).

Figure S10. ^{1}H-NMR spectra of compound **TR4**

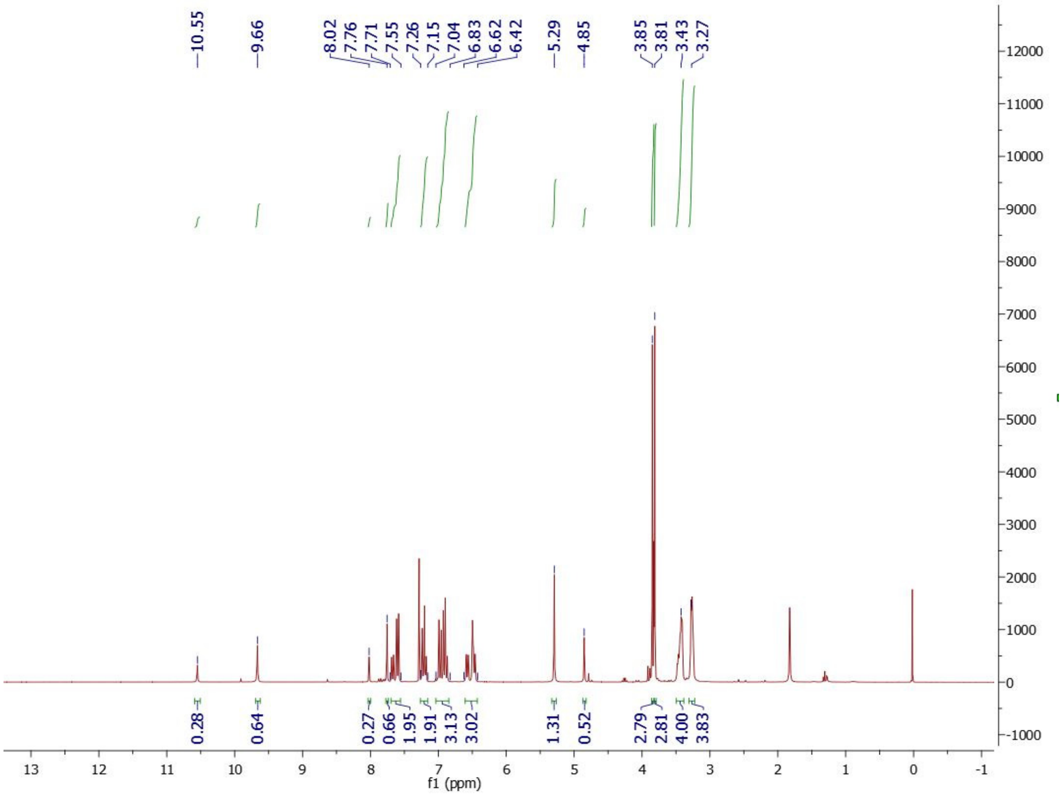

8

Figure S11. ^{13}C-NMR spectra of compound **TR4**

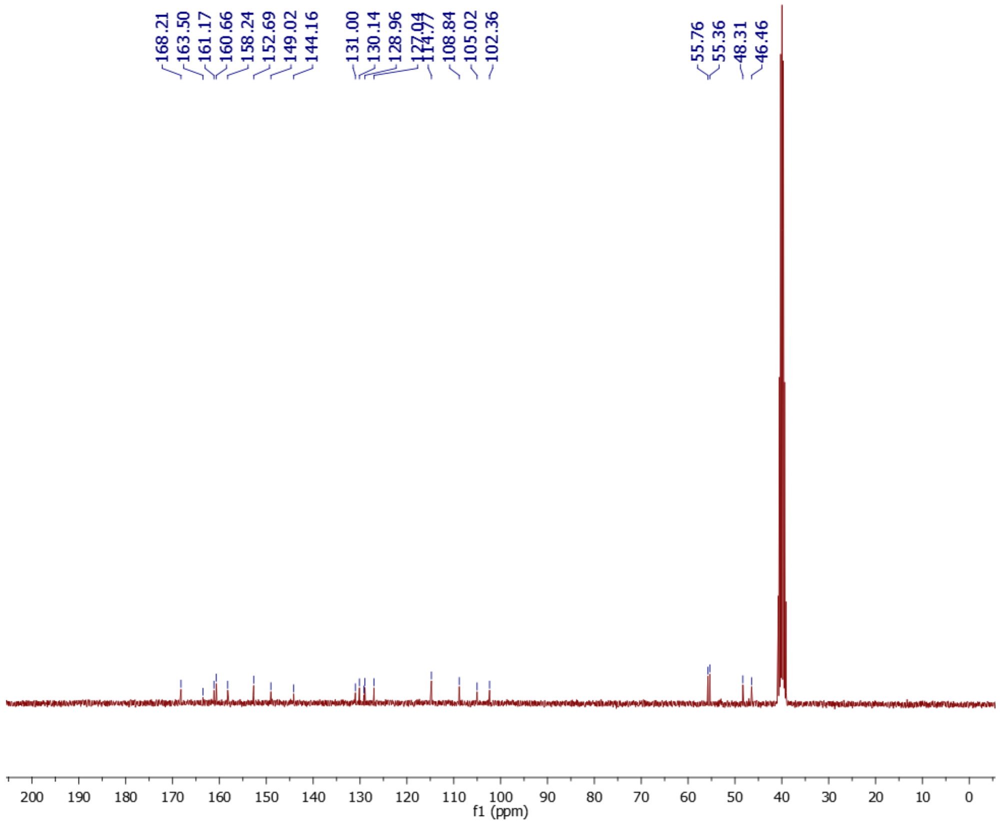

Figure S12. HRMS spectra of compound **TR4**

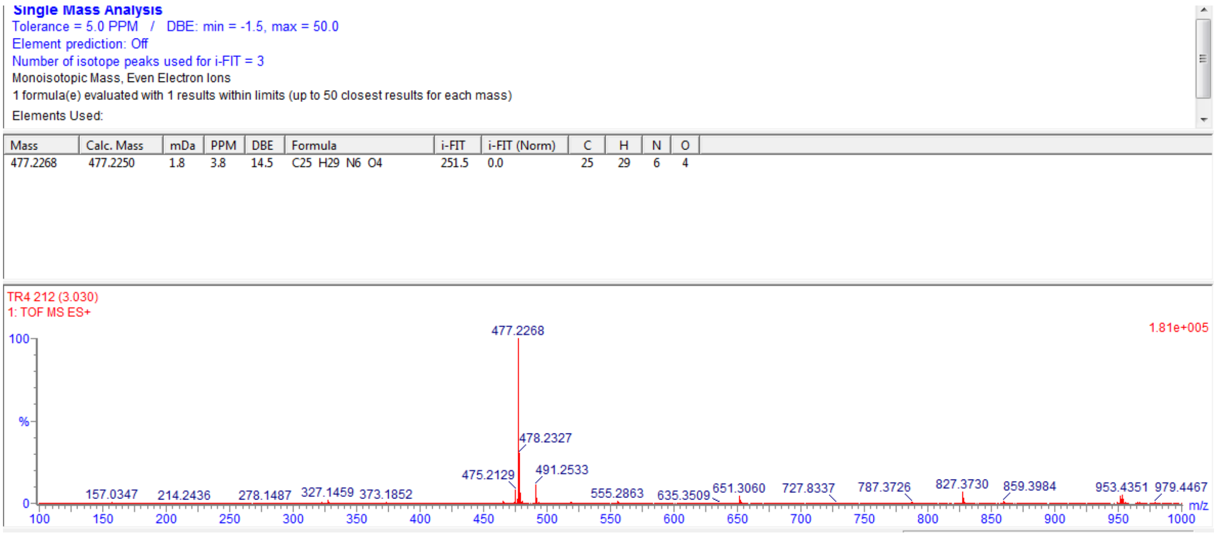

N'-(4-methoxybenzylidene)-2-(3-(4-(3-methoxyphenyl)piperazin-1-yl)-6-oxopyridazin-1(6H)-yl)acetohydrazide (**TR4**)

^{1}H-NMR (DMSO-d_6, 300 MHz): δ 3.25 (4H; t; CH2N; b+b′), 3.42 (4H; t; CH2N; a+a′), 3.81 (3H; s; OCH3), 5.29 (2H; s; CH2CO), 6.45-7.69 (10H; m; phenyl protons, pyridazinone $H^{4,5}$), 8.02 (1H; s; -N=CH-) and 9.66 (1H; s; -NH-N).

^{13}C-NMR (DMSO-d_6, 300 MHz), δ 46.46 (2C, CH_2-N; b+b′), 48.31 (2C, CH_2-N; a+a′), 55.36 (1C; -N-$\underline{C}H_2$-C=O), 55.76 (2C, OCH_3), 102.36 (1C; =CH), 105.02 (2C; 4-methoxyphenyl $C^{3,5}$), 108.84 (2C; 4-methoxyphenyl $C^{2,6}$), 114.77 (1C; 4-methoxyphenyl C^1), 127.04 (1C; 3-methoxyphenyl C^5), 128.96 (1C; pyridazinone C^4), 130.14 (1C; 3-methoxyphenyl C^6), 131.00 (1C; 3-methoxyphenyl C^1), 144.16 (1C; 3-methoxyphenyl C^4), 149.02 (1C; 3-methoxyphenyl C^2), 152.69 (1C; pyridazinone C^5), 158.24 (1C; pyridazinone C^6), 160.66 (1C; 4-methoxyphenyl C^4), 161.17 (1C; 3-methoxyphenyl C^3), 163.50 (1C; CH_2-N-$\underline{C}$=O), 168.21 (1C; pyridazinone C^3);

$C_{25}H_{29}N_6O_4$ MS (ESI+) calculated: 477.2250, Found: m/e 477.2268 (M^+; 100.0%).

Figure S13. ^{1}H-NMR spectra of compound **TR5**

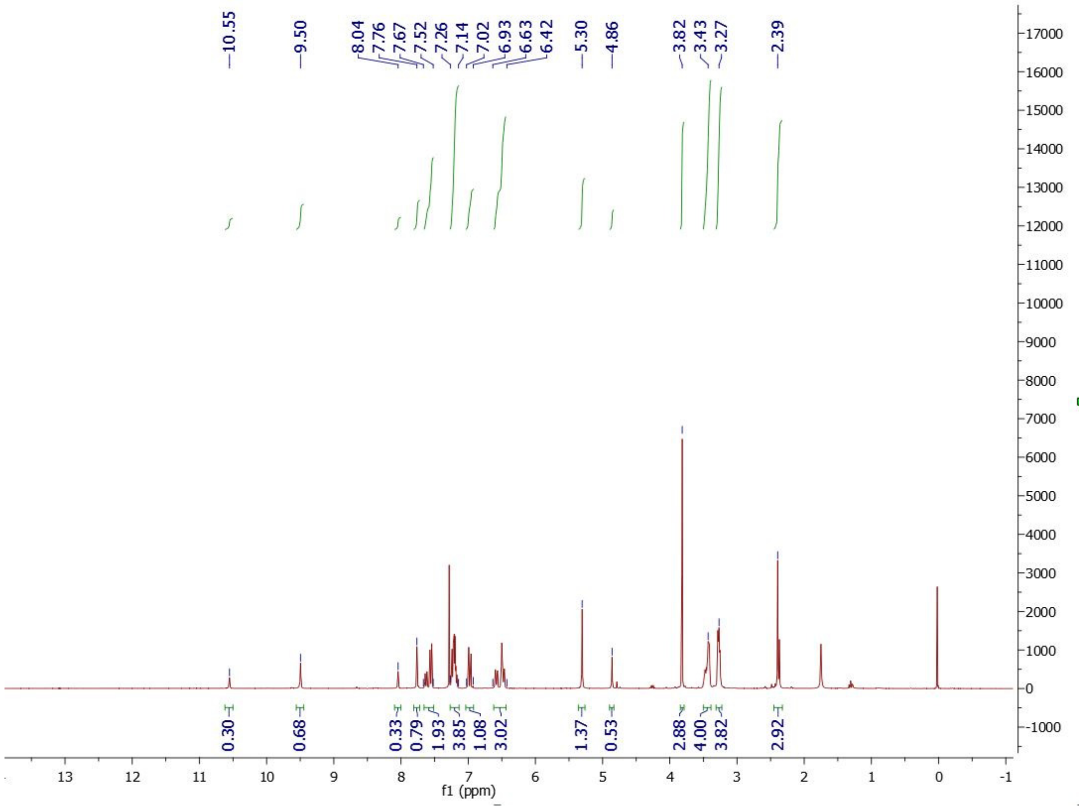

Figure S14. ^{13}C-NMR spectra of compound **TR5**

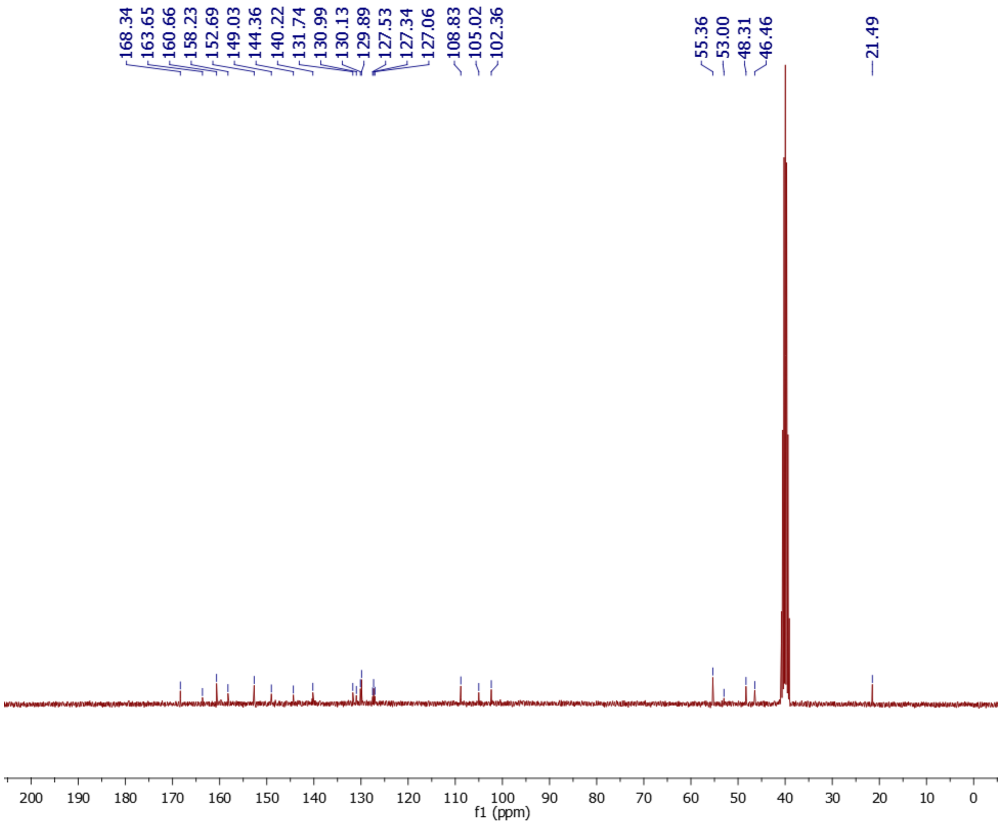

Figure S15. HRMS spectra of compound **TR5**

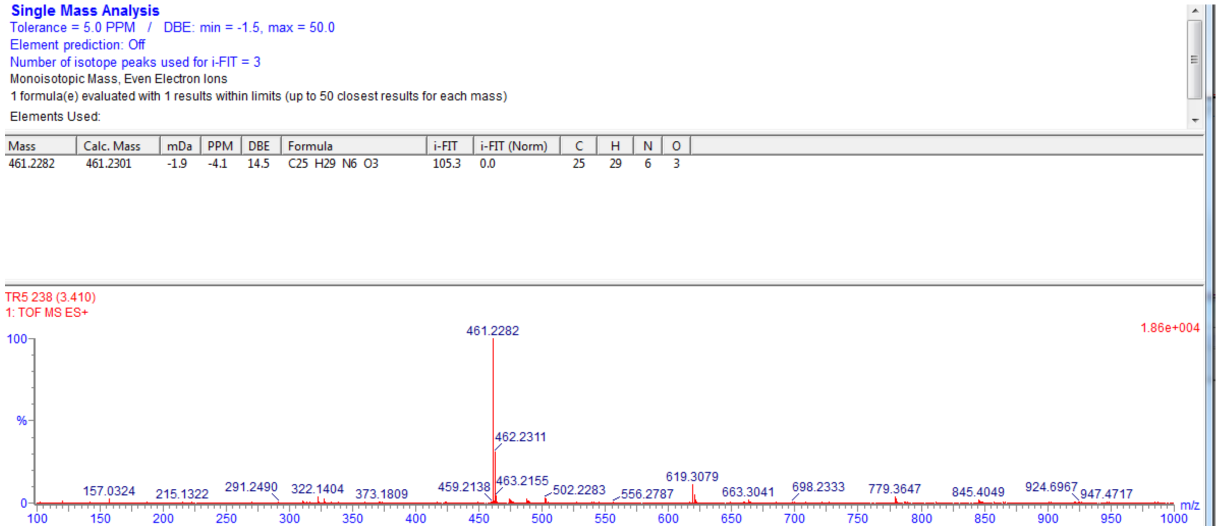

N'-(4-methylbenzylidene)-2-(3-(4-(3-methoxyphenyl)piperazin-1-yl)-6-oxopyridazin-1(6H)-yl)acetohydrazide (**TR5**)

^{1}H-NMR (DMSO-d_6, 300 MHz): δ 2.39 (3H; d; -CH_3), 3.25 (4H; t; CH_2N; b+b'), 3.42 (4H; t; CH_2N; a+a'), 3.82 (3H; s; OCH3), 5.30 (2H; s; CH_2CO), 6.45-7.64 (10H; m; phenyl protons, pyridazinone $H^{4,5}$), 8.04 (1H; s; -N=CH-) and 9.59 (1H; s; -NH-N).

^{13}C-NMR (DMSO-d$_6$, 300 MHz), δ 21.49 (1C; -CH$_3$), 46.46 (2C, CH$_2$-N; b+b′), 48.31 (2C, CH$_2$-N; a+a′), 53.00 (1C; -N-C̲H$_2$-C=O), 55.36 (1C, -OCH$_3$), 102.36 (1C; =CH), 105.02 (1C; 4-methylphenyl C^4), 108.83 (1C; 4-methylphenyl C^3), 127.06 (1C; 4-methylphenyl C^5), 127.34 (1C; 4-methylphenyl C^2), 127.53 (1C; 4-methylphenyl C^6), 129.89 (1C; 4-methylphenyl C^1), 130.13 (1C; 3-methoxyphenyl C^5), 130.99 (1C; pyridazinone C^4), 131.74 (1C; 3-methoxyphenyl C^6), 140.22 (1C; 3-methoxyphenyl C^1), 144.36 (1C; 3-methoxyphenyl C^4), 149.03 (1C; 3-methoxyphenyl C^2), 152.69 (1C; pyridazinone C^5), 158.23 (1C; pyridazinone C^6), 160.66 (1C; 3-methoxyphenyl C^3), 163.65 (1C; CH$_2$-N-C̲=O), 168.34 (1C; pyridazinone C^3);

$C_{25}H_{29}N_6O_3$ MS (ESI+) calculated: 461.2301, Found: m/e 461.2282 (M$^+$; 100.0%).

Figure S16. ^{1}H-NMR spectra of compound **TR6**

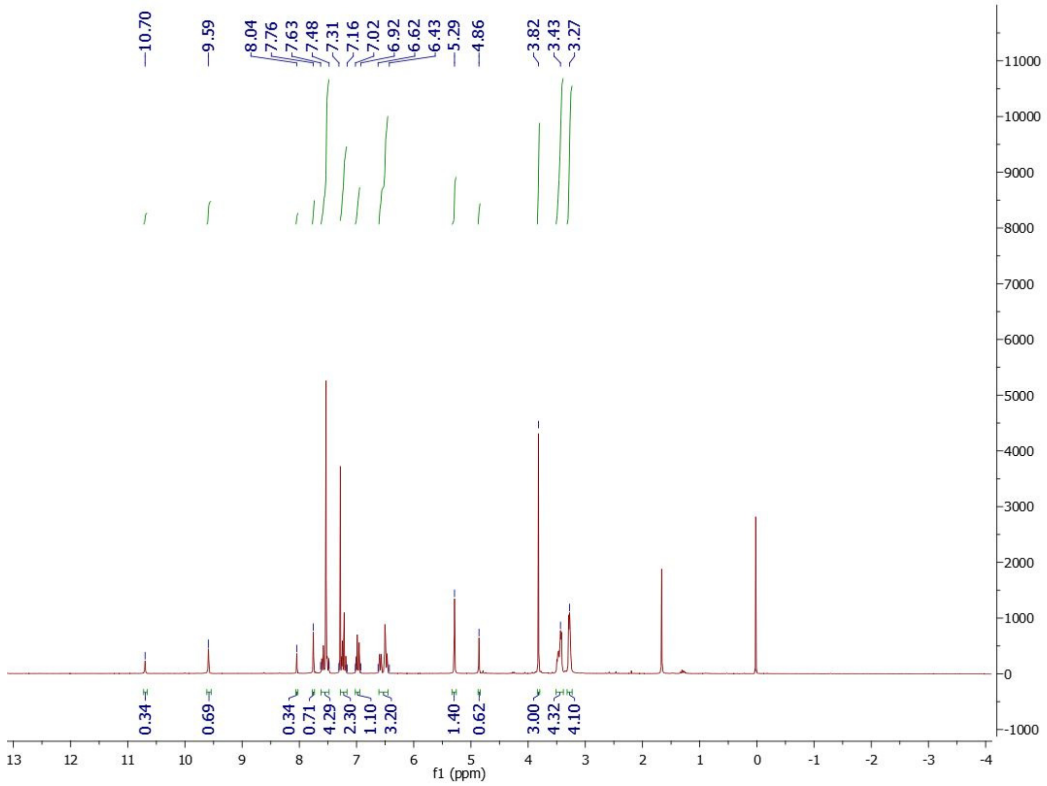

Figure 17. ^{13}C-NMR spectra of compound **TR6**

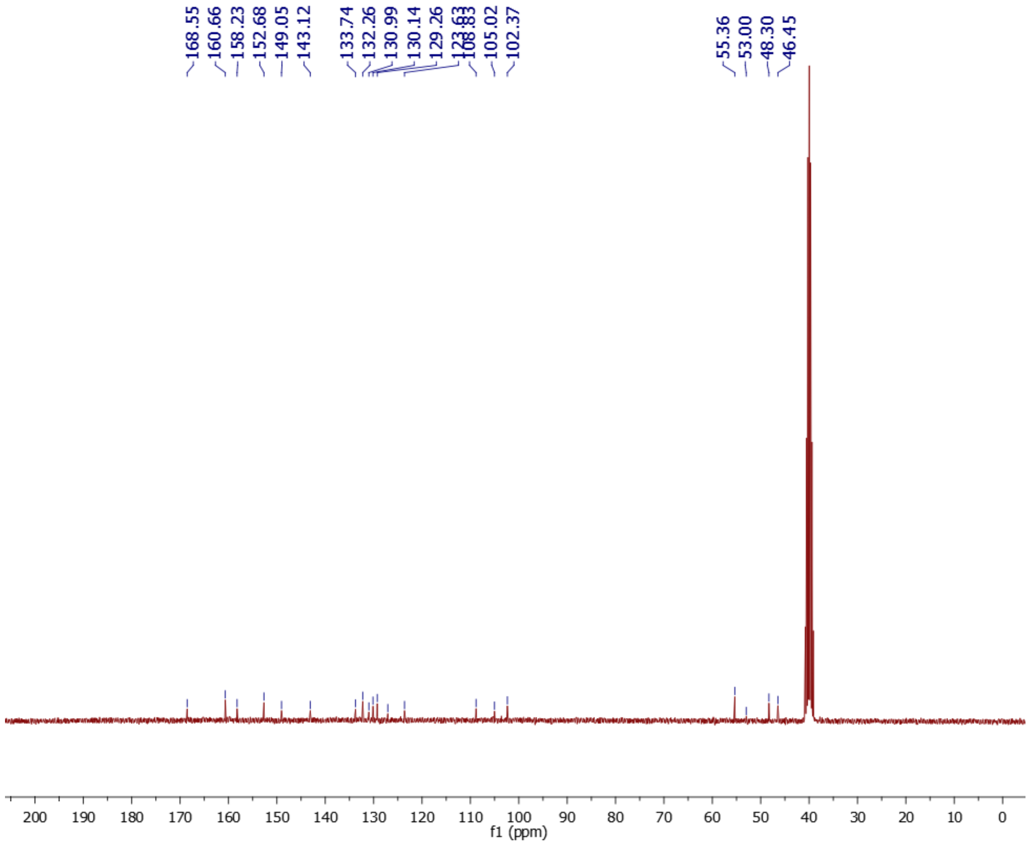

Figure S18. HRMS spectra of compound **TR6**

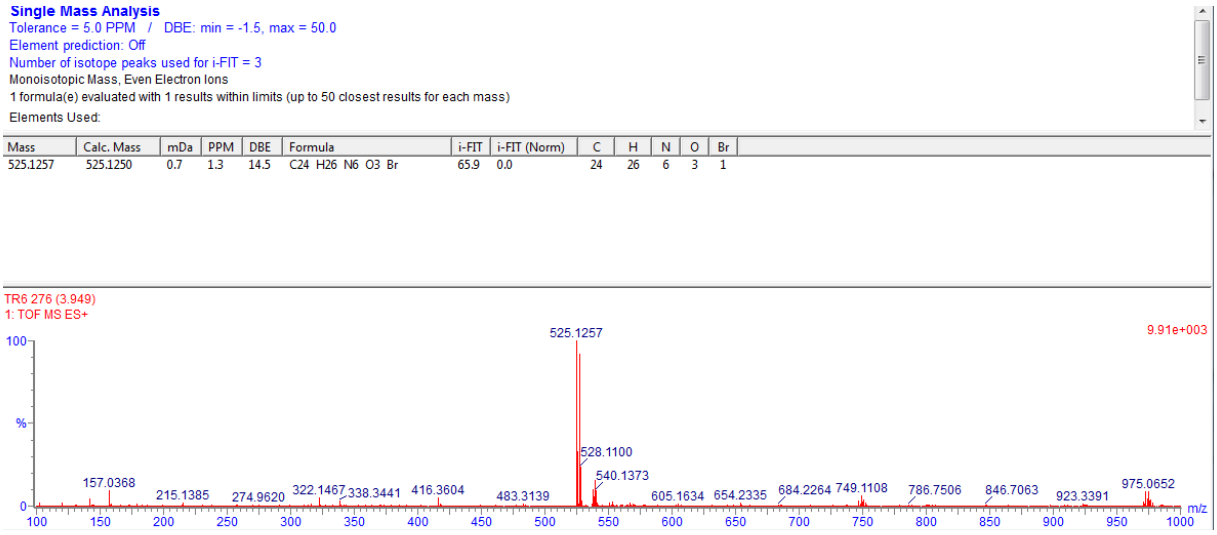

N'-(4-bromobenzylidene)-2-(3-(4-(3-methoxyphenyl)piperazin-1-yl)-6-oxopyridazin-1(6H)-yl)acetohydrazide (**TR6**)

^{1}H-NMR (DMSO-d_6, 300 MHz): δ 3.26 (4H; t; CH_2N; b+b'), 3.43 (4H; t; CH_2N; a+a'), 3.82 (3H; s; $-OCH_3$), 5.29 (2H; s; CH_2CO), 6.43-7.60 (10H; m; phenyl protons, pyridazinone $H^{4,5}$), 8.04 (1H; s; -N=CH-) and 9.59 (1H; s; -NH-N).

13

^{13}C-NMR (DMSO-d_6, 300 MHz), δ 46.45 (2C, CH_2-N; b+b'), 48.30 (2C, CH_2-N; a+a'), 53.00 (1C; -N-$\underline{C}H_2$-C=O), 55.36 (1C, OCH3), 102.37 (1C; =CH), 105.02 (2C; 4-bromophenyl $C^{3,5}$), 108.83 (2C; 4-bromophenyl $C^{2,6}$), 123.63 (1C; 4-bromophenyl C^1), 129.26 (1C; pyridazinone C^4), 130.14 (2C; 3-methoxyphenyl $C^{5,6}$), 130.99 (1C; 3-methoxyphenyl C^1), 132.26 (1C; 3-methoxyphenyl C^4), 133.74 (1C; 3-methoxyphenyl C^2), 143.12 (1C; pyridazinone C^5), 149.05 (1C; pyridazinone C^6), 152.68 (1C; 4-bromophenyl C^4), 158.23 (1C; 3-methoxyphenyl C^3), 160.66 (1C; CH_2-N-$\underline{C}$=O), 168.55 (1C; pyridazinone C^3);

$C_{24}H_{26}BrN_6O_3$ MS (ESI+) calculated: 525.1250, Found: m/e 525.1257 (M^+; 100.0%).

Figure 19. ^{1}H-NMR spectra of compound **TR7**

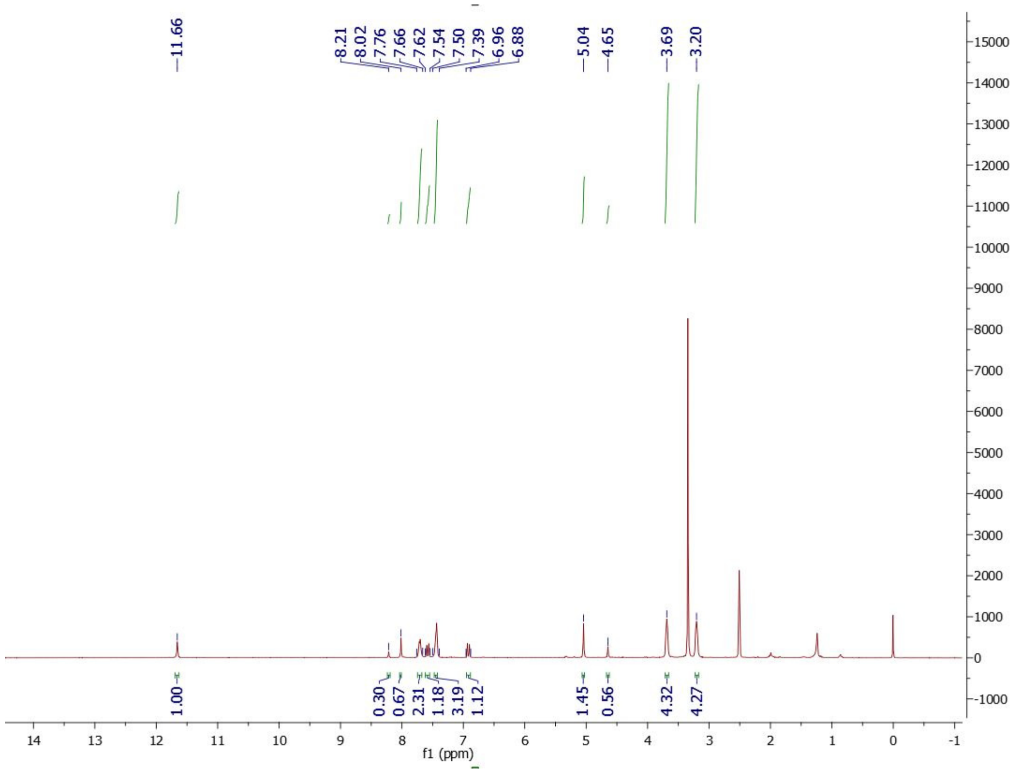

Figure S20. ^{13}C-NMR spectra of compound **TR7**

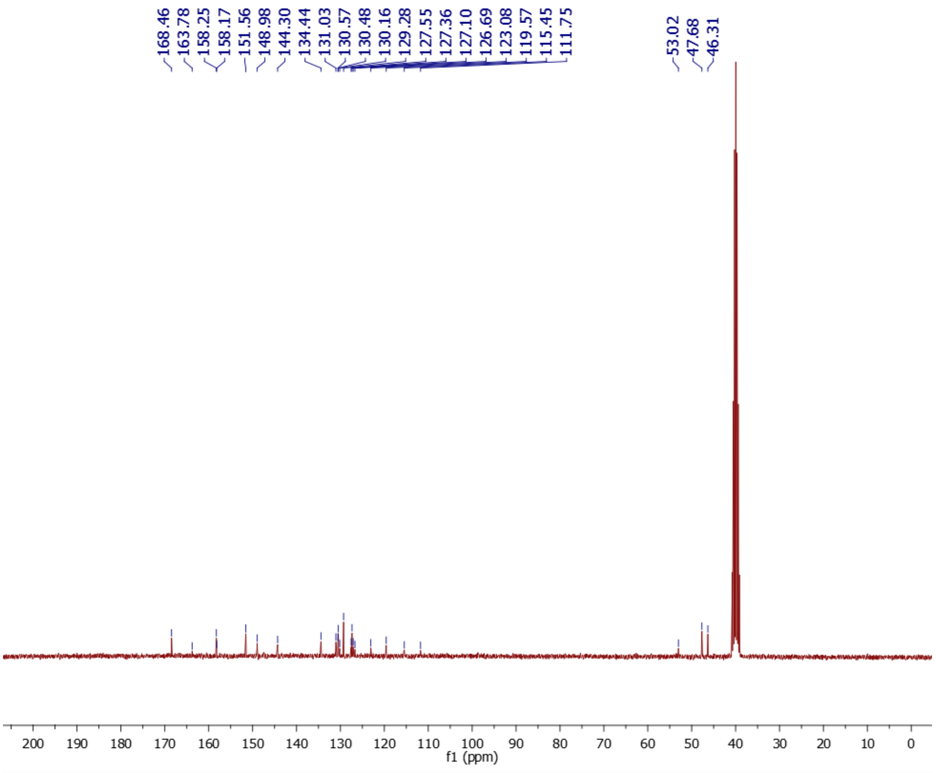

Figure 21. HRMS spectra of compound **TR7**

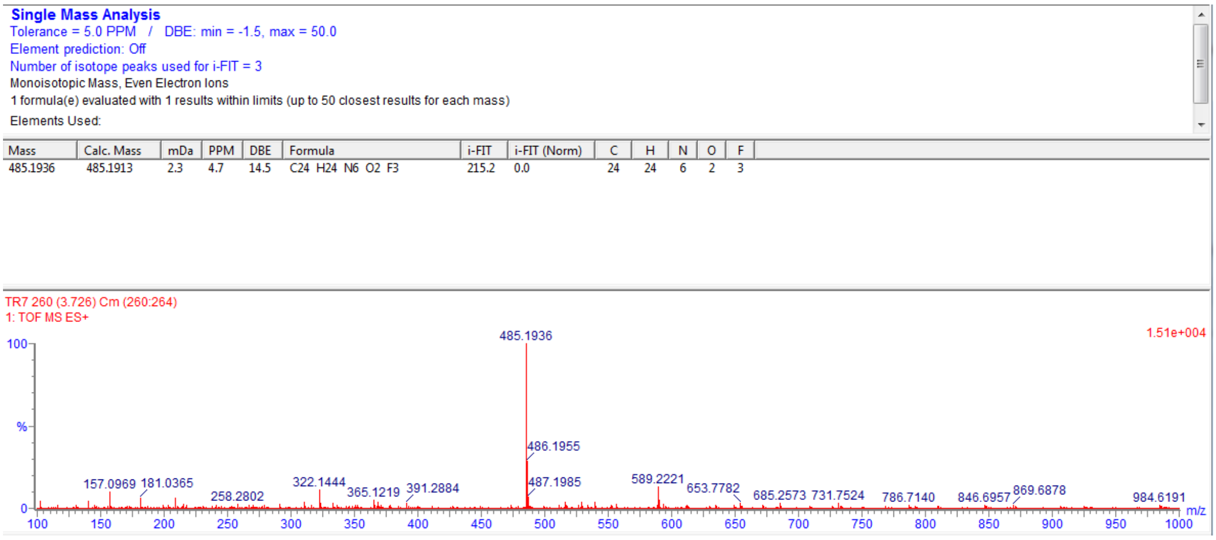

N'-benzylidene-2-(3-(4-(3-trifloromethylphenyl)piperazin-1-yl)-6-oxopyridazin-1(6H)-yl)acetohydrazide (**TR7**)

^{1}H-NMR (DMSO-d_6, 300 MHz): δ 3.18 (8H; t; CH_2N; b+b′, a+a′), 5.07 (2H; s; CH_2CO), 6.68-7.77 (11H; m; phenyl protons, pyridazinone $H^{4,5}$), 8.22 (1H; s; -N=CH-) and 11.67 (1H; s; -NH-N).

^{13}C-NMR (DMSO-d_6, 300 MHz), δ 46.31 (2C, CH_2-N; b+b′), 47.68 (2C, CH_2-N; a+a′), 53.02 (1C; -N-<u>C</u>H_2-C=O), 111.75, 115.45 (1C; -CF_3), 119.57 (1C; =CH), 123.08 (1C; phenyl

15

C^3), 126.69 (1C; phenyl C^5), 127.10 (1C; phenyl C^2), 127.36 (1C; phenyl C^6), 127.55 (1C; phenyl C^1), 129.28 (1C; phenyl C^4), 130.16 (1C; pyridazinone C^4), 130.48 (1C; 3-trifluoromethylphenyl C^5), 130.57 (1C; 3-trifluoromethylphenyl C^6), 131.03 (1C; 3-trifluoromethylphenyl C^4), 134.44 (1C; 3-trifluoromethylphenyl C^2), 144.30 (1C; 3-trifluoromethylphenyl C^1), 148.98, 151.56 (1C; 3-trifluoromethylphenyl C^3), 158.17 (1C; pyridazinone C^5), 158.25 (1C; pyridazinone C^6), 163.78 (1C; CH_2-N-$\underline{C}$=O), 168.46 (1C; pyridazinone C^3);

$C_{24}H_{24}F_3N_6O_2$ MS (ESI+) calculated: 485.1916, Found: m/e 485.1936 (M^+; 100.0%).

Figure S22. ^{1}H-NMR spectra of compound **TR8**

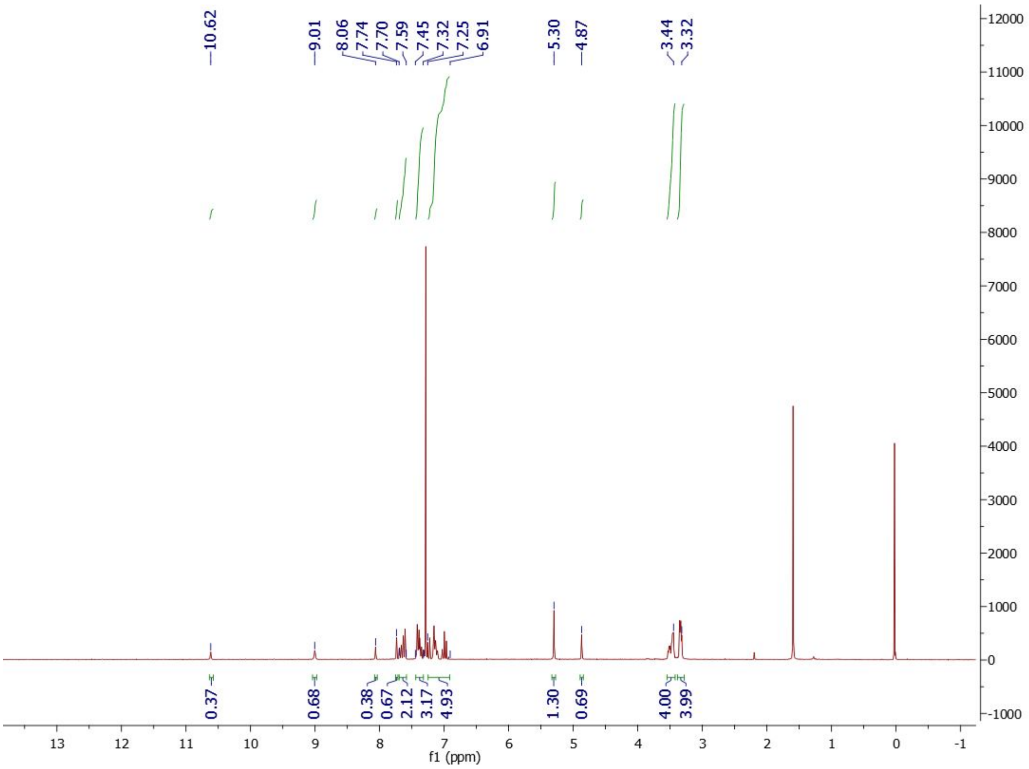

Figure S23. ^{13}C-NMR spectra of compound **TR8**

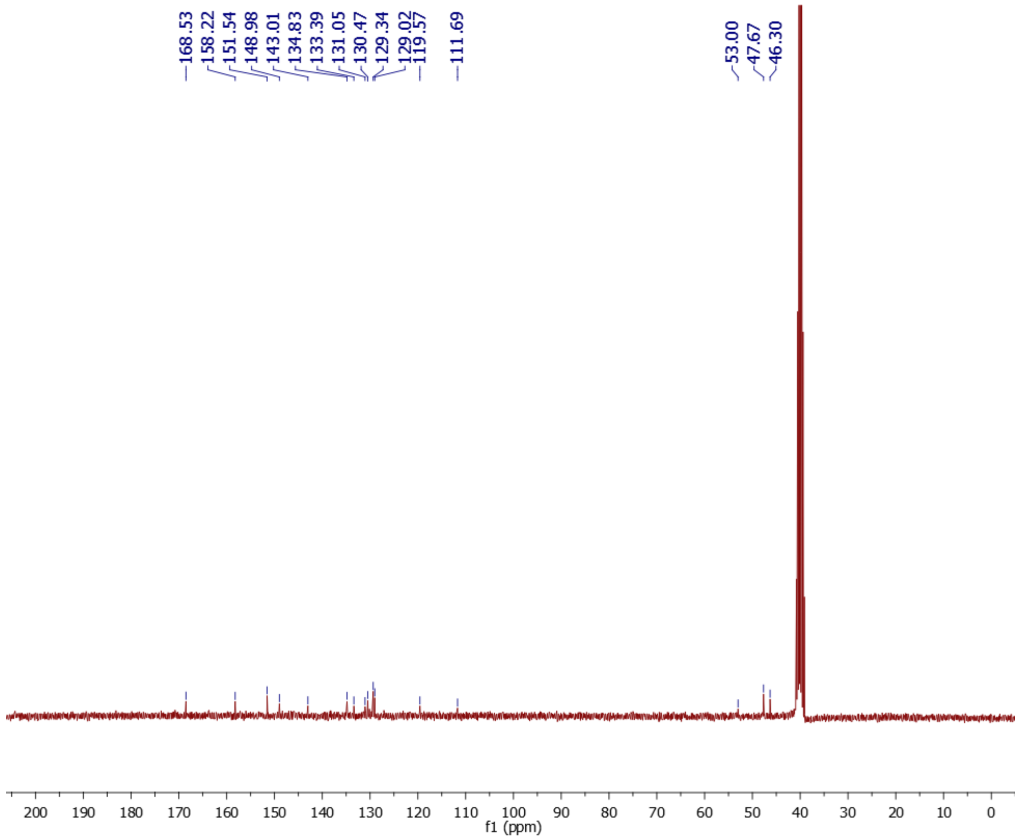

Figure S24. HRMS spectra of compound **TR8**

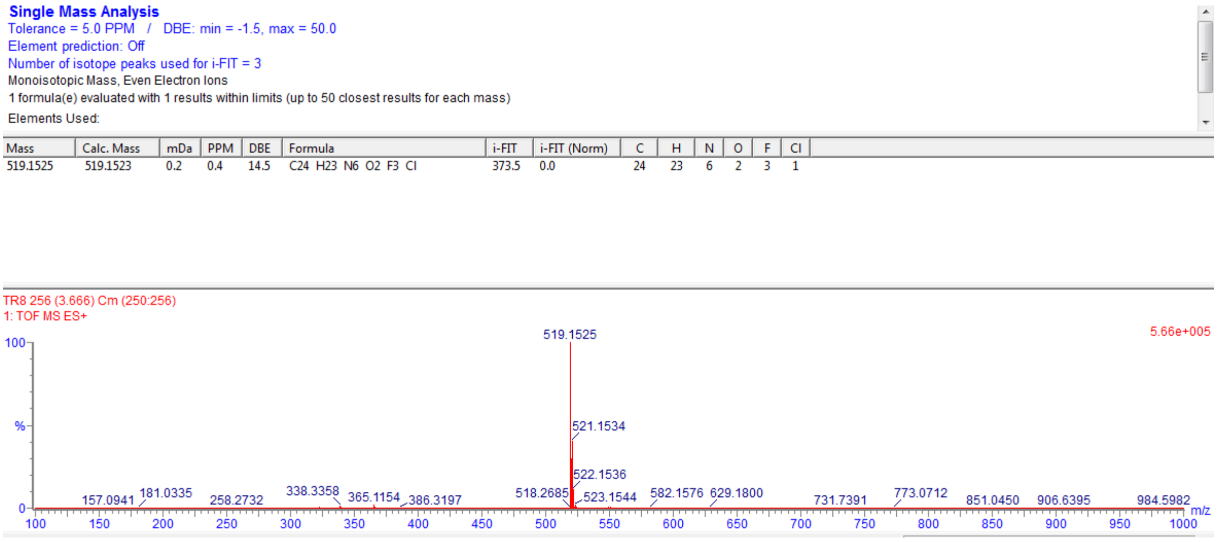

Mass	Calc. Mass	mDa	PPM	DBE	Formula	i-FIT	i-FIT (Norm)	C	H	N	O	F	Cl
519.1525	519.1523	0.2	0.4	14.5	C24 H23 N6 O2 F3 Cl	373.5	0.0	24	23	6	2	3	1

N'-(4-chlorobenzylidene)-2-(3-(4-(3-trifloromethylphenyl)piperazin-1-yl)-6-oxopyridazin-1(6H)-yl)acetohydrazide (**TR8**)

^{1}H-NMR (DMSO-d$_6$, 300 MHz): δ 3.34 (4H; t; CH_2N; b+b’), 3.46 (4H; t; CH_2N; a+a’), 5.30 (2H; s; CH_2CO), 6.96-7.69 (10H; m; phenyl protons, pyridazinone H^{4,5}), 9.01 (1H; s; -N=CH-) and 10.62 (1H; s; -NH-N).

^{13}C-NMR (DMSO-d$_6$, 300 MHz), δ 46.30 (2C, CH$_2$-N; b+b'), 47.67 (2C, CH$_2$-N; a+a'), 53.00 (1C; -N-C̲H$_2$-C=O), 111.69 (1C; -CF$_3$), 119.57 (1C; =CH), 129.02 (2C; 4-clorophenyl C^{3,5}), 129.34 (2C; 4-clorophenyl C^{2,6}), 130.47 (1C; 4-clorophenyl C^{1}), 131.05 (1C; pyridazinone C^{4}), 133.39 (4C; 3-trifluoromethylphenyl C^{2,4,5,6}), 134.83 (1C; 3-trifluoromethylphenyl C^{1}), 143.01 (1C; 3-trifluoromethylphenyl C^{3}), 148.98 (2C; pyridazinone C^{5,6}), 151.54 (1C; 4-clorophenyl C^{4}), 158.22 (1C; CH$_2$-N-C̲=O), 168.53 (1C; pyridazinone C^{3});

$C_{24}H_{23}ClF_3N_6O_2$ MS (ESI+) calculated: 519.1523, Found: m/e 519.1525 (M$^+$; 100.0%).

Figure S25. ^{1}H-NMR spectra of compound **TR9**

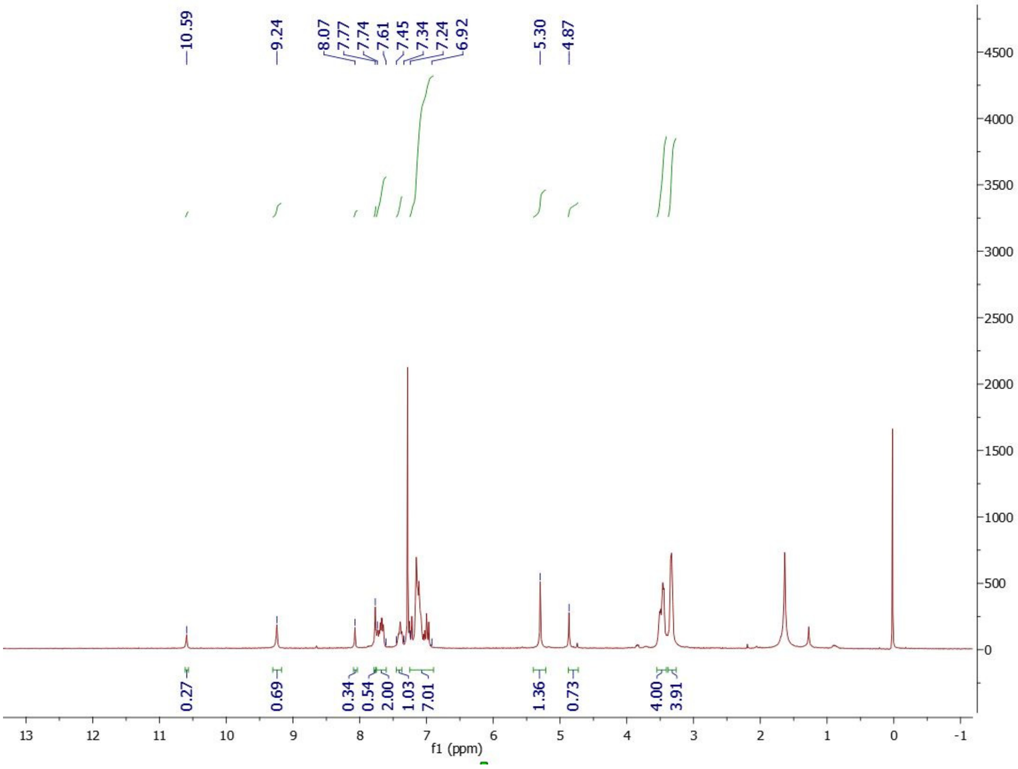

Figure S26. ^{13}C-NMR spectra of compound **TR9**

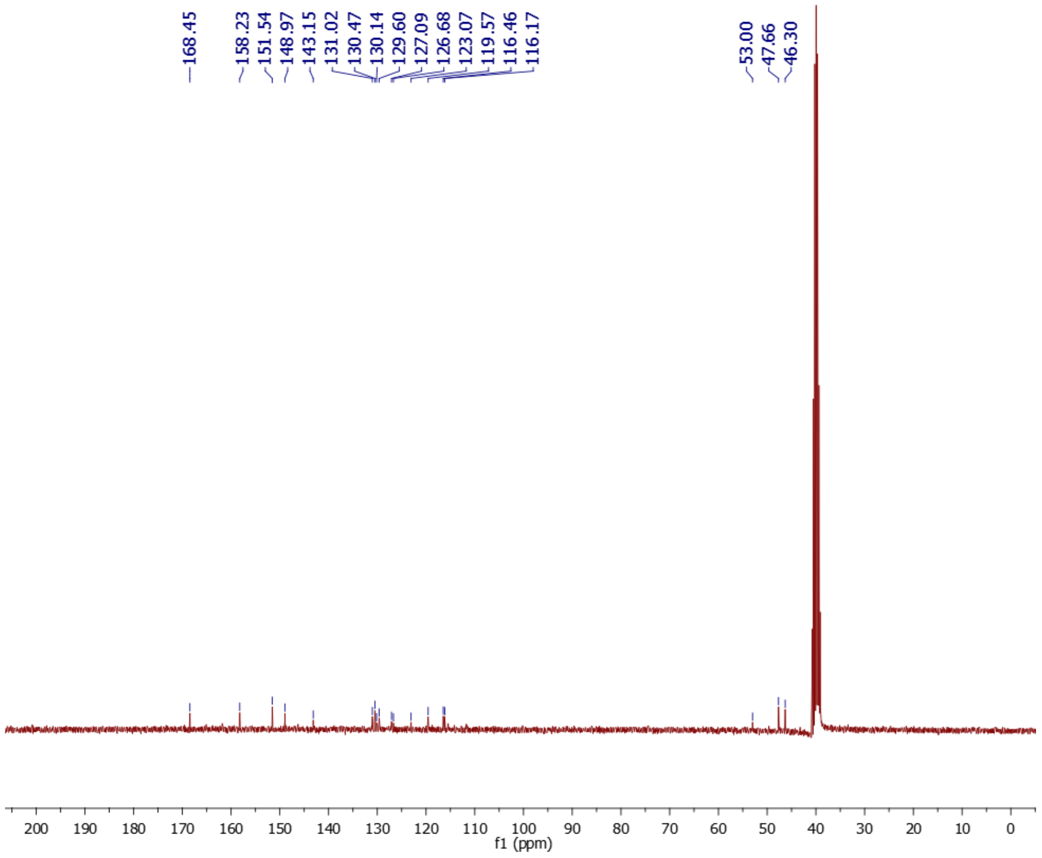

Figure S27. HRMS spectra of compound **TR9**

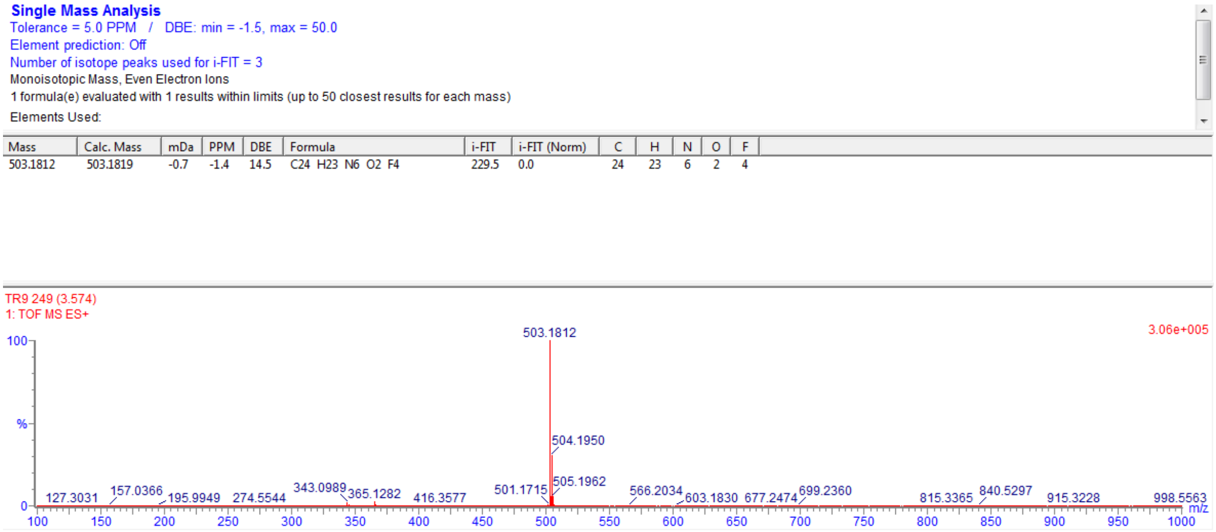

N'-(4-florobenzylidene)-2-(3-(4-(3-trifloromethylphenyl)piperazin-1-yl)-6-oxopyridazin-1(6H)-yl)acetohydrazide (**TR9**)

^{1}H-NMR (DMSO-d_6, 300 MHz): δ 3.33 (4H; t; CH_2N; b+b'), 3.45 (4H; t; CH_2N; a+a'), 5.30 (2H; s; CH_2CO), 6.97-7.74 (10H; m; phenyl protons, pyridazinone $H^{4,5}$), 8.07 (1H; s; -N=CH-) and 10.59 (1H; s; -NH-N).

^{13}C-NMR (DMSO-d_6, 300 MHz), δ 46.30 (2C, CH_2-N; b+b'), 47.66 (2C, CH_2-N; a+a'), 53.00 (1C; -N-CH_2-C=O), 116.17 (1C; -CF_3), 116.46 (1C; =CH), 119.57 (2C; 4-fluorophenyl $C^{3,5}$), 123.07 (2C; 4-fluorophenyl $C^{2,6}$), 126.68 (1C; 4-fluorophenyl C^1), 127.09 (1C; pyridazinone C^4), 129.60 (3C; 3-trifluoromethylphenyl $C^{4,5,6}$), 130.14 (1C; 3-trifluoromethylphenyl C^2), 130.47 (1C; 3-trifluoromethylphenyl C^1), 131.02 (1C; 3-trifluoromethylphenyl C^3), 143.15 (1C; pyridazinone C^5), 148.97 (1C; pyridazinone C^6), 151.54 (1C; 4-fluorophenyl C^4), 158.23 (1C; CH_2-N-C=O), 168.45 (1C; pyridazinone C^3);

$C_{24}H_{23}F_4N_6O_2$ MS (ESI+) calculated: 503.1819, Found: m/e 503.1812 (M^+; 100.0%).

Figure S28. ^{1}H-NMR spectra of compound **TR10**

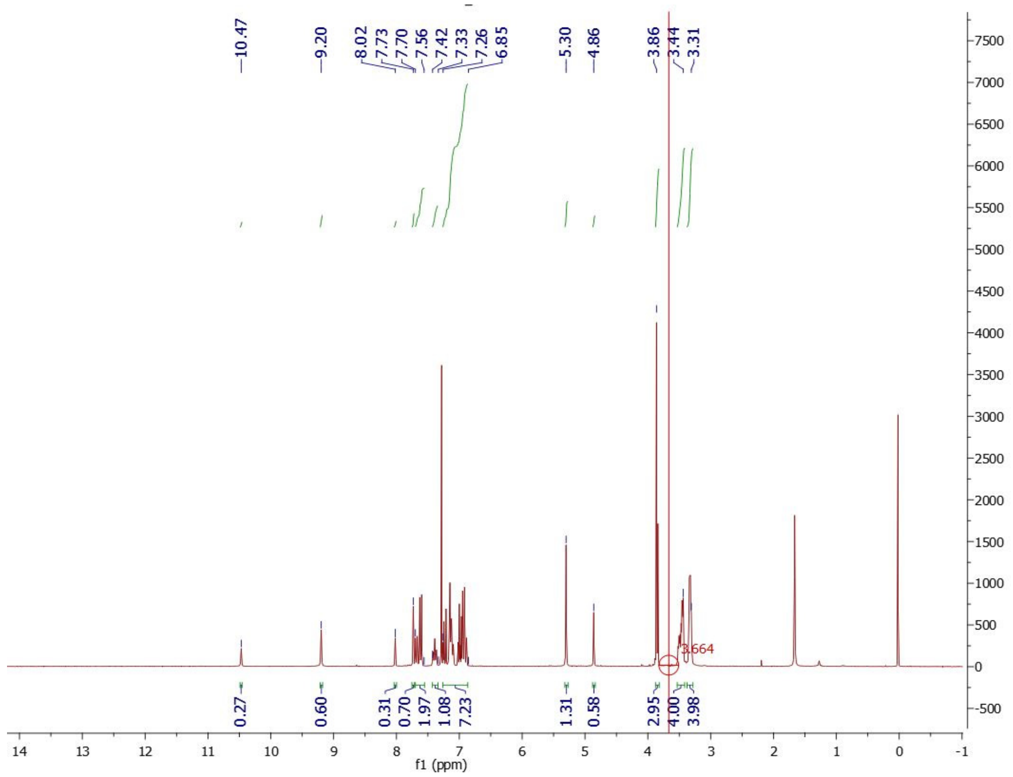

Figure S29. ^{13}C-NMR spectra of compound **TR10**

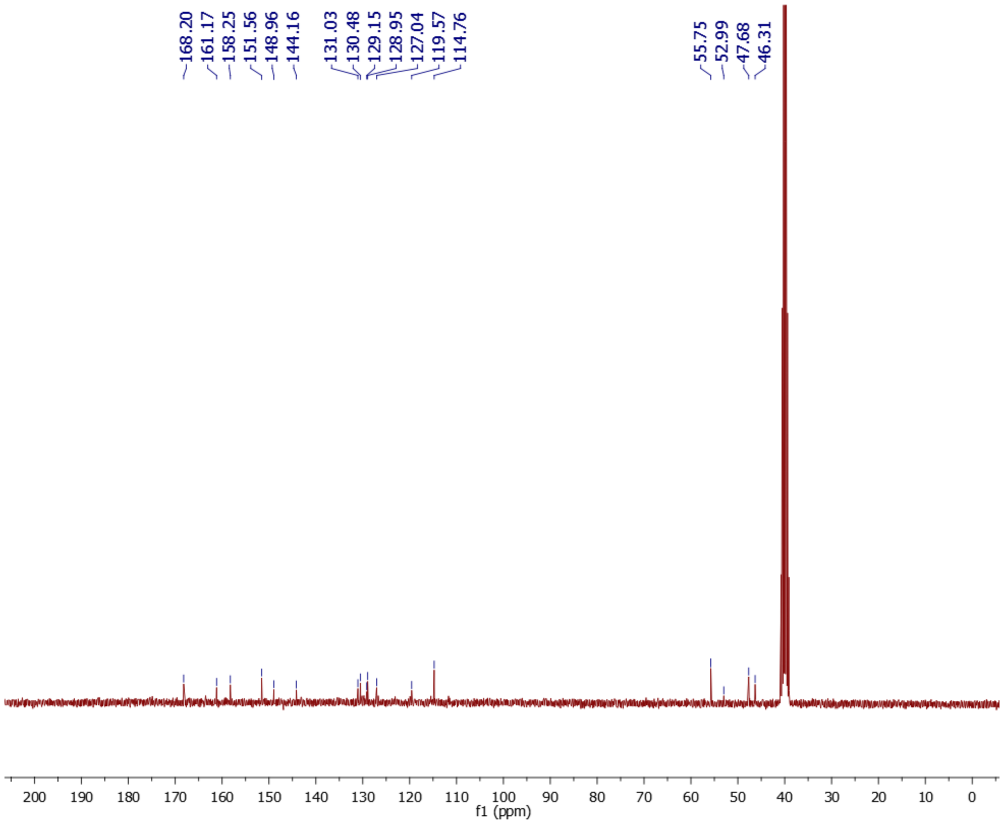

Figure S30. HRMS spectra of compound **TR10**

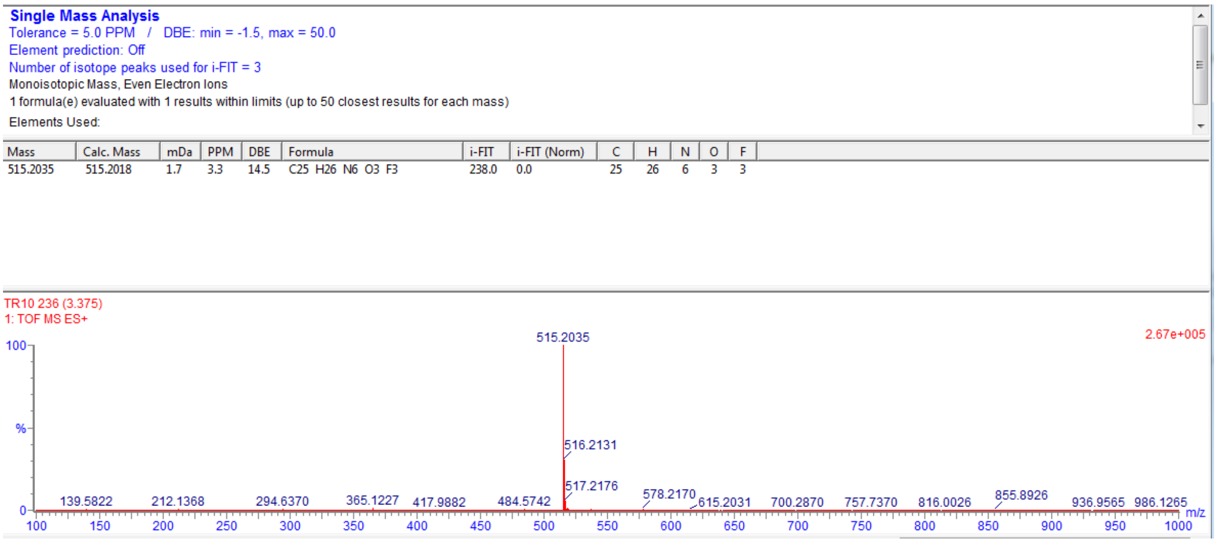

N'-(4-methoxybenzylidene)-2-(3-(4-(3-trifloromethylphenyl)piperazin-1-yl)-6-oxopyridazin-1(6H)-yl)acetohydrazide (**TR10**)

^{1}H-NMR (DMSO-d$_6$, 300 MHz): δ 3.34 (4H; t; CH_2N; b+b'), 3.46 (4H; t; CH_2N; a+a'), 3.84 (3H; s; -OCH_3), 5.30 (2H; s; CH_2CO), 6.88-7.35 (10H; m; phenyl protons, pyridazinone $H^{4,5}$), 8.02 (1H; s; -N=CH-) and 10.47 (1H; s; -NH-N).

^{13}C-NMR (DMSO-d$_6$, 300 MHz), δ 46.31 (2C, CH$_2$-N; b+b'), 47.68 (2C, CH$_2$-N; a+a'), 52.99 (1C; -N-C̲H$_2$-C=O), 55.75 (1C; -OCH$_3$), 114.76 (1C; -CF$_3$), 119.57 (1C; =CH), 127.04 (2C; 4-methoxyphenyl C^{3,5}), 128.95 (2C; 4-methoxyphenyl C^{2,6}), 129.15 (1C; 4-methoxyphenyl C^{1}), 130.48 (1C; pyridazinone C^{4}), 131.03 (4C; 3-trifluoromethylphenyl C^{2,4,5,6}), 144.16 (1C; 3-trifluoromethylphenyl C^{1}), 148.96 (1C; 3-trifluoromethylphenyl C^{3}), 151.56 (2C; pyridazinone C^{5,6}), 158.25 (1C; 4-methoxyphenyl C^{4}), 161.17 (1C; CH$_2$-N-C̲=O), 168.20 (1C; pyridazinone C^{3});

$C_{25}H_{26}F_3N_6O_3$ MS (ESI+) calculated: 515.2018, Found: m/e 515.2035 (M$^+$; 100.0%).

Figure S31. ^{1}H-NMR spectra of compound **TR11**

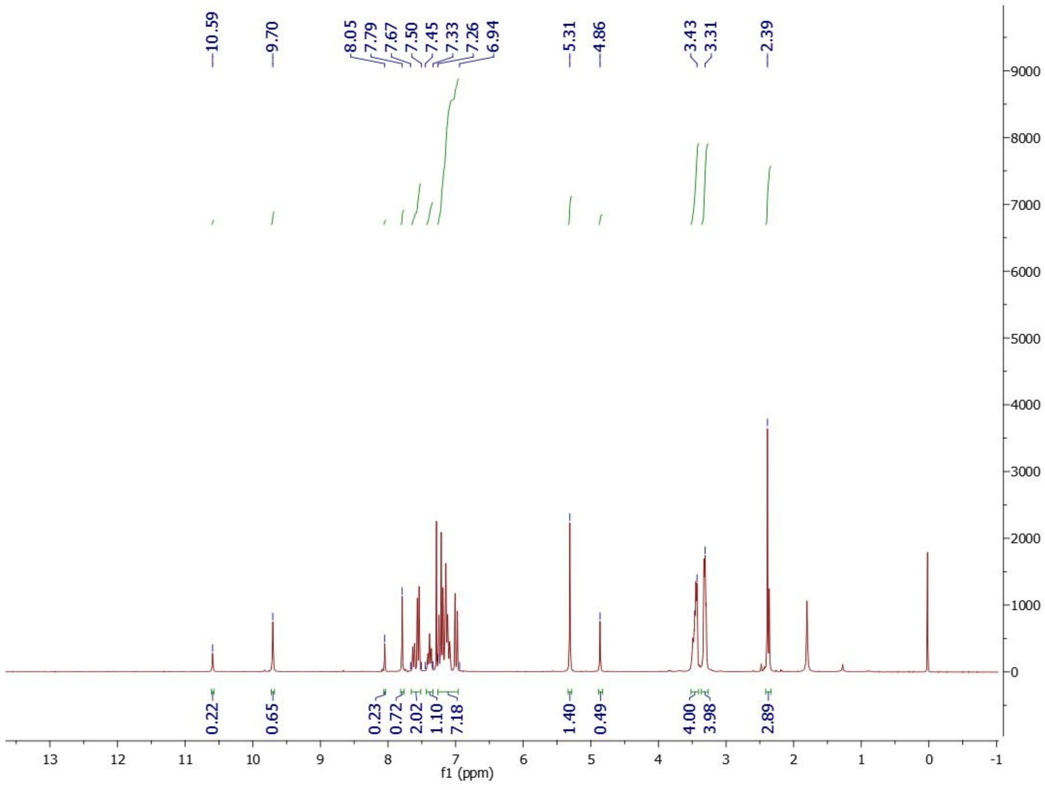

Figure S32. ^{13}C-NMR spectra of compound **TR11**

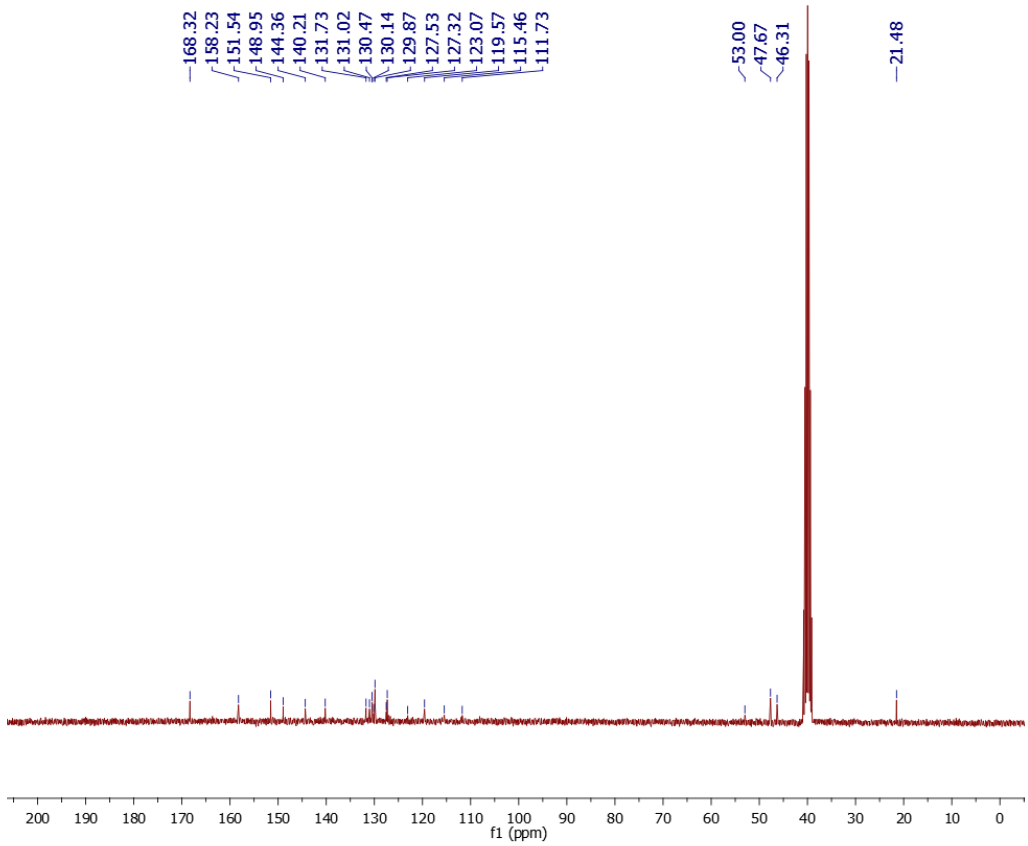

Figure S33. HRMS spectra of compound **TR11**

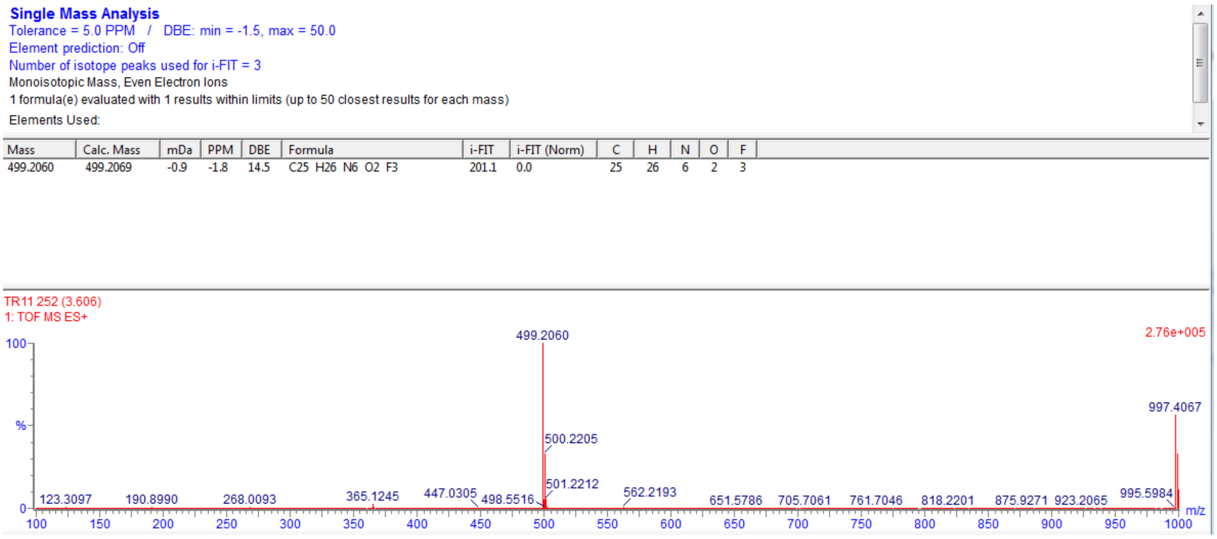

N'-(4-methylbenzylidene)-2-(3-(4-(3-trifloromethylphenyl)piperazin-1-yl)-6-oxopyridazin-1(6H)-yl)acetohydrazide (**TR11**)

^{1}H-NMR (DMSO-d_6, 300 MHz): δ 2.39 (3H; d; -CH_3), 3.31 (4H; t; CH_2N; b+b'), 3.43 (4H; t; CH_2N; a+a'), 5.31 (2H; s; CH_2CO), 6.97-7.63 (10H; m; phenyl protons, pyridazinone $H^{4,5}$), 9.70 (1H; s; -N=CH-) and 10.59 (1H; s; -NH-N).

^{13}C-NMR (DMSO-d$_6$, 300 MHz), δ 21.48 (1C; -CH$_3$), 46.31 (2C, CH$_2$-N; b+b'), 47.67 (2C, CH$_2$-N; a+a'), 53.00 (1C; -N-C̲H$_2$-C=O), 111.73 (1C; -CF$_3$), 115.46 (1C; =CH), 119.57 (1C; 4-methylphenyl C^4), 123.07 (2C; 4-methylphenyl C^{3,5}), 127.32 (2C; 4-methylphenyl C^{2,6}), 127.53 (1C; 4-methylphenyl C^1), 129.87 (1C; pyridazinone C^4), 130.14 (2C; 3-trifluoro-methylphenyl C^{4,5}), 130.47 (1C; 3-trifluoromethylphenyl C^6), 131.02 (1C; 3-trifluoro-methylphenyl C^2), 131.73 (1C; 3-trifluoromethylphenyl C^1), 144.36 (1C; 3-trifluoro-methylphenyl C^3), 148.95 (1C; pyridazinone C^5), 151.54 (1C; pyridazinone C^6), 158.23 (1C; CH$_2$-N-C̲=O), 168.32 (1C; pyridazinone C^3);

$C_{25}H_{26}F_3N_6O_2$ MS (ESI+) calculated: 499.2069, Found: m/e 499.2060 (M$^+$; 100.0%).

Figure S34. ^{1}H-NMR spectra of compound **TR12**

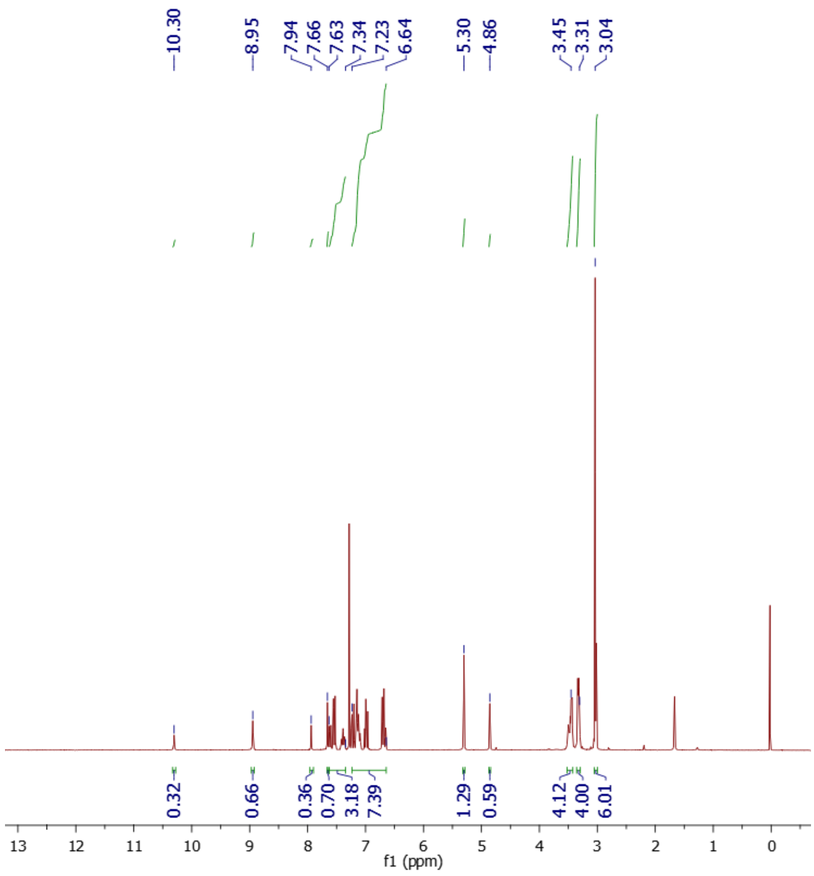

Figure S35. ^{13}C-NMR spectra of compound **TR12**

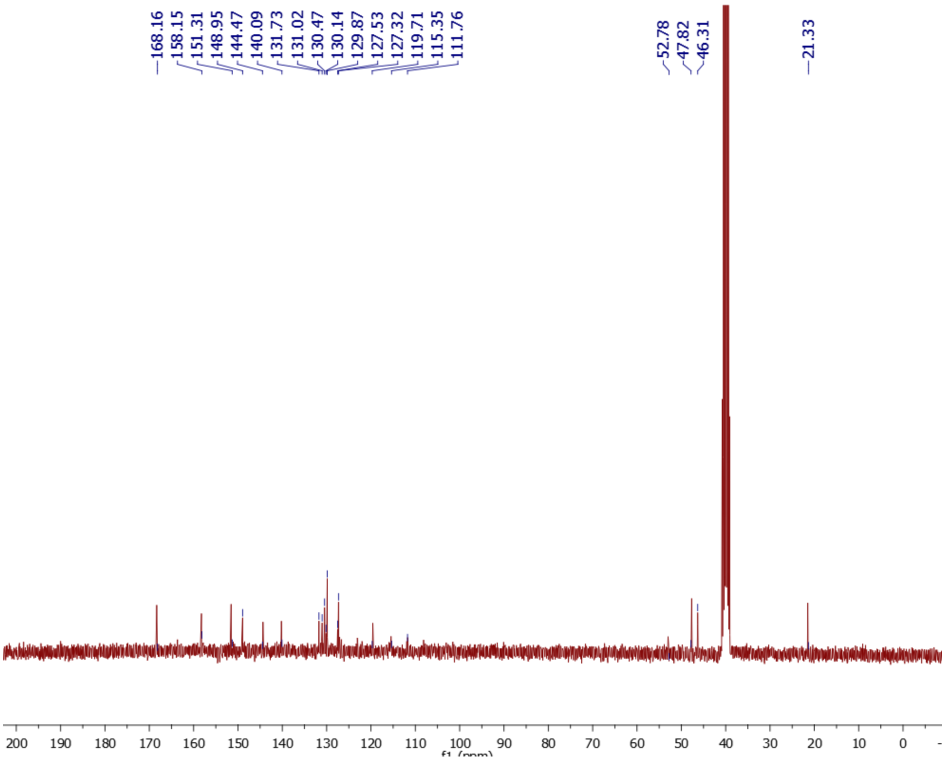

Figure S36. HRMS spectra of compound **TR12**

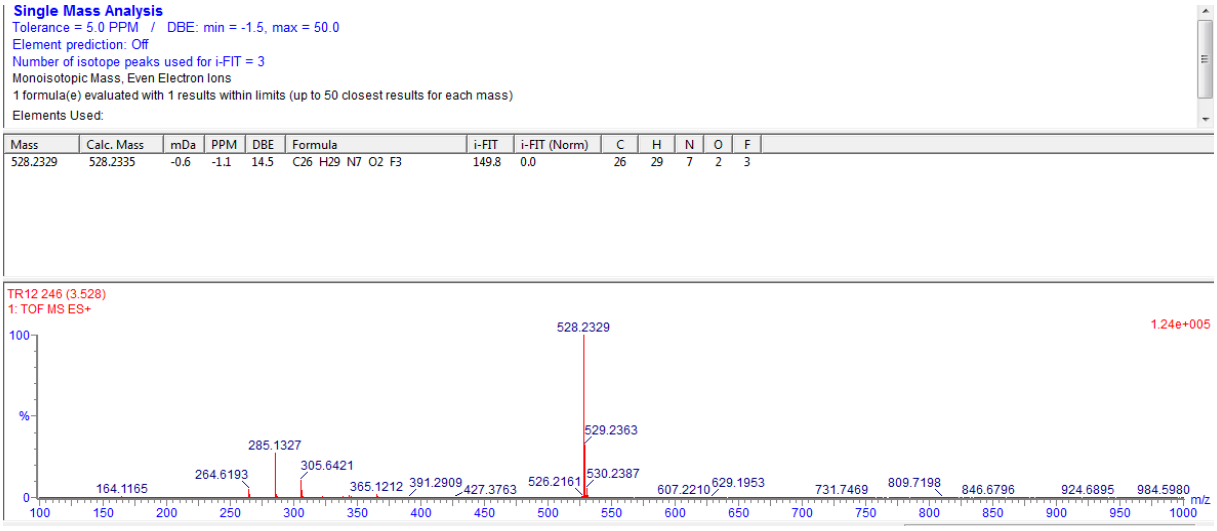

N'-(4-dimethylaminobenzylidene)-2-(3-(4-(3-trifloromethylphenyl)piperazin-1-yl)-6-oxopyridazin-1(6H)-yl)acetohydrazide (**TR12**)

^{1}H-NMR (DMSO-d$_6$, 300 MHz): δ 3.04 (6H; s; N(CH$_3$)$_2$), 3.31 (4H; t; CH$_2$N; b+b'), 3.45 (4H; t; CH$_2$N; a+a'), 5.30 (2H; s; CH$_2$CO), 6.64-7.94 (10H; m; phenyl protons, pyridazinone H^{4,5}), 8.95 (1H; s; -N=CH-) and 10.30 (1H; s; -NH-N).

^{13}C-NMR (DMSO-d$_6$, 300 MHz), δ 21.33 (2C; N(CH$_3$)$_2$), 46.31 (2C, CH$_2$-N; b+b'), 47.82 (2C, CH$_2$-N; a+a'), 52.78 (1C; -N-C̲H$_2$-C=O), 111.76 (1C; -CF$_3$), 119.71 (1C; =CH), 127.32 (2C;

4-dimethylaminophenyl $C^{3,5}$), 127.53 (2C; 4-dimethylaminophenyl $C^{2,6}$), 129.87 (1C; 4-dimethylaminophenyl C^{1}), 130.47 (1C; pyridazinone C^{4}), 131.02 (4C; 3-trifluoromethylphenyl $C^{2,4,5,6}$), 131.73 (1C; 3-trifluoromethylphenyl C^{1}), 140.09 (1C; 3-trifluoromethylphenyl C^{3}), 144.47 (1C; pyridazinone C^{5}), 148.95 (1C; pyridazinone C^{6}), 151.31 (1C; 4-dimethylaminophenyl C^{4}), 158.15 (1C; CH_2-N-C=O), 168.16 (1C; pyridazinone C^{3});

$C_{26}H_{29}F_3N_7O_2$ MS (ESI+) calculated: 528.2335, Found: m/e 528.2329 (M^+; 100.0%).

Figure S37. ^{1}H-NMR spectra of compound **TR13**

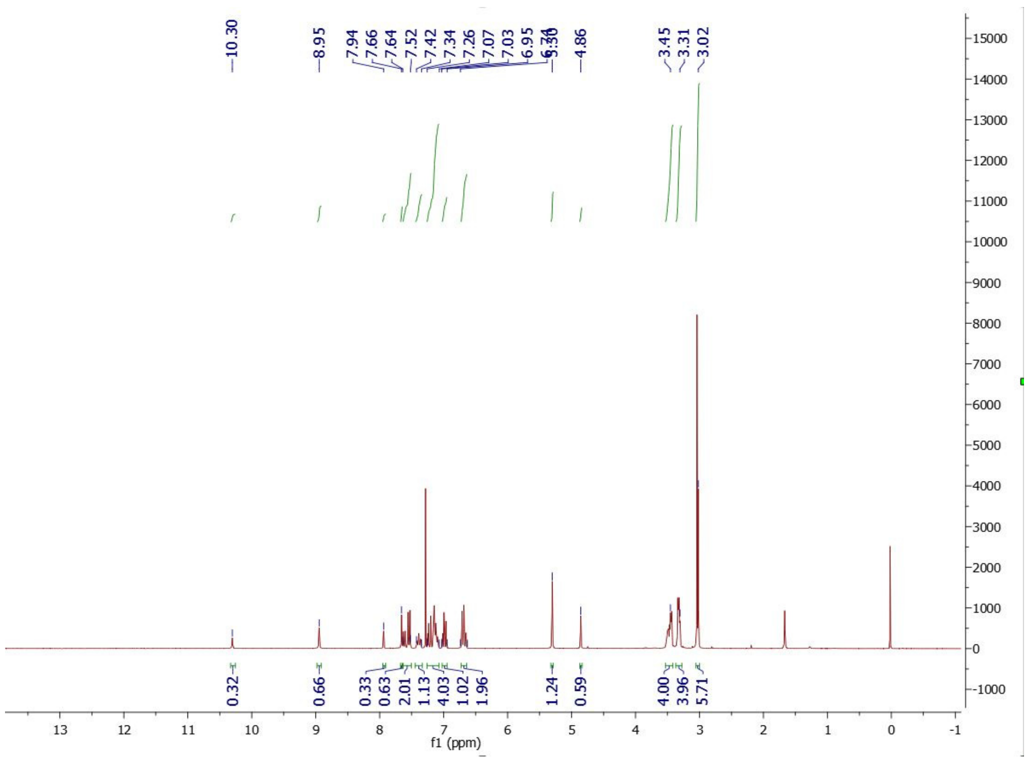

Figure S38. ^{13}C-NMR spectra of compound **TR13**

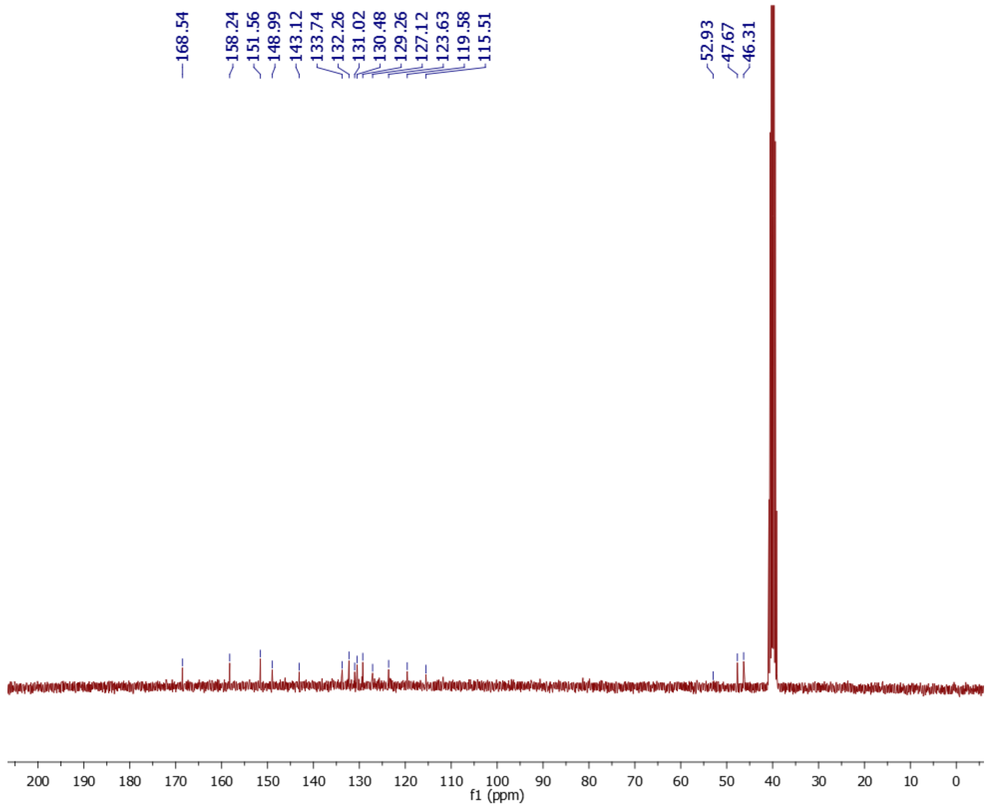

Figure S39. HRMS spectra of compound **TR13**

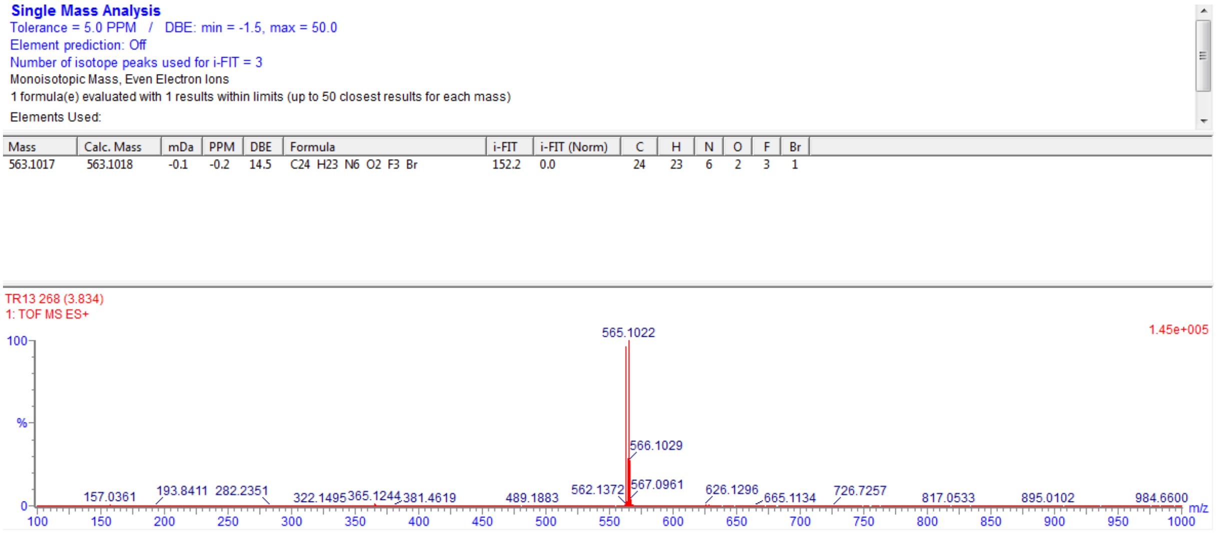

N'-(4-bromobenzylidene)-2-(3-(4-(3-trifloromethylphenyl)piperazin-1-yl)-6-oxopyridazin-1(6H)-yl)acetohydrazide (**TR13**)

^{1}H-NMR (DMSO-d$_6$, 300 MHz): δ 3.32 (4H; t; CH_2N; b+b'), 3.45 (4H; t; CH_2N; a+a'), 5.30 (2H; s; CH_2CO), 6.93-7.45 (9H; m; phenyl protons, pyridazinone H^{4,5}), 8.98 (1H; s; -N=CH-) and 10.62 (1H; s; -NH-N).

^{13}C-NMR (DMSO-d$_6$, 300 MHz), δ 46.31 (2C, CH$_2$-N; b+b'), 47.67 (2C, CH$_2$-N; a+a'), 52.93 (1C; -N-C̲H$_2$-C=O), 115.51 (1C; -CF$_3$), 119.58 (1C; =CH), 123.63 (2C; 4-bromophenyl C^{3,5}), 127.12 (2C; 4-bromophenyl C^{2,6}), 129.26 (1C; 4-bromophenyl C^{1}), 130.48 (1C; pyridazinone C^{4}), 131.02 (4C; 3-trifluoromethylphenyl C^{2,4,5,6}), 132.26 (1C; 3-trifluoromethylphenyl C^{1}), 133.74 (1C; 3-trifluoromethylphenyl C^{3}), 143.12 (1C; pyridazinone C^{5}), 148.99 (1C; pyridazinone C^{6}), 151.56 (1C; 4-bromophenyl C^{4}), 158.24 (1C; CH$_2$-N-C̲=O), 168.54 (1C; pyridazinone C^{3});

$C_{24}H_{23}BrF_3N_6O_2$ MS (ESI+) calculated: 563.1018, Found: m/e 563.1017 (M$^+$; 100.0%).

Figure S40. ^{1}H-NMR spectra of compound **TR14**

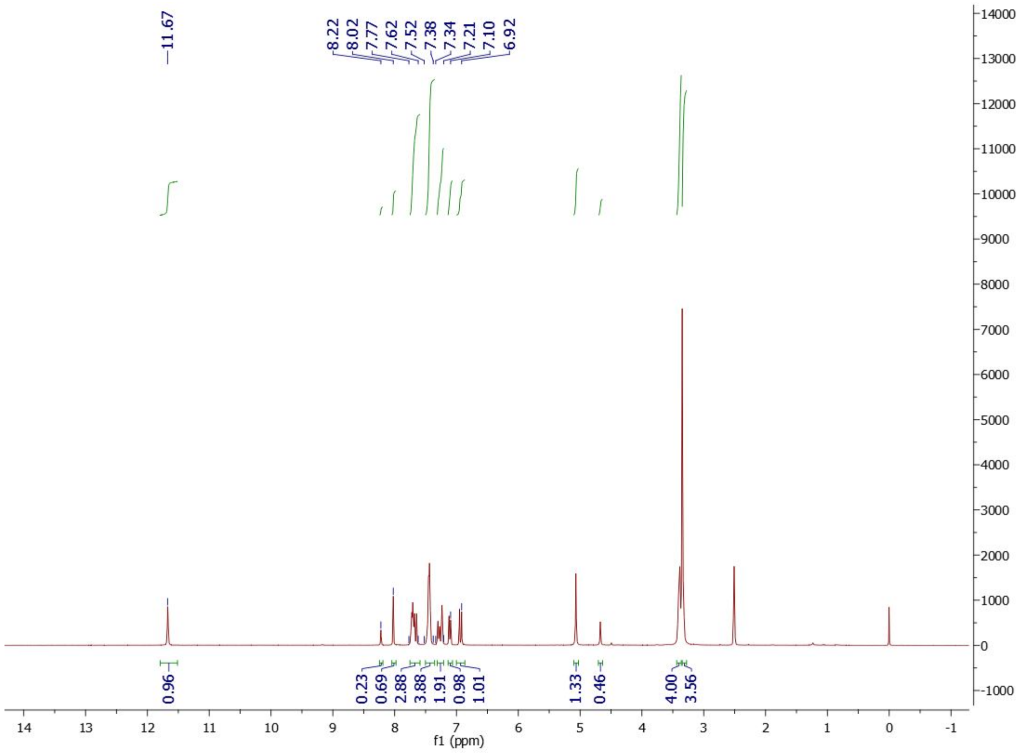

Figure S41. ^{13}C-NMR spectra of compound **TR14**

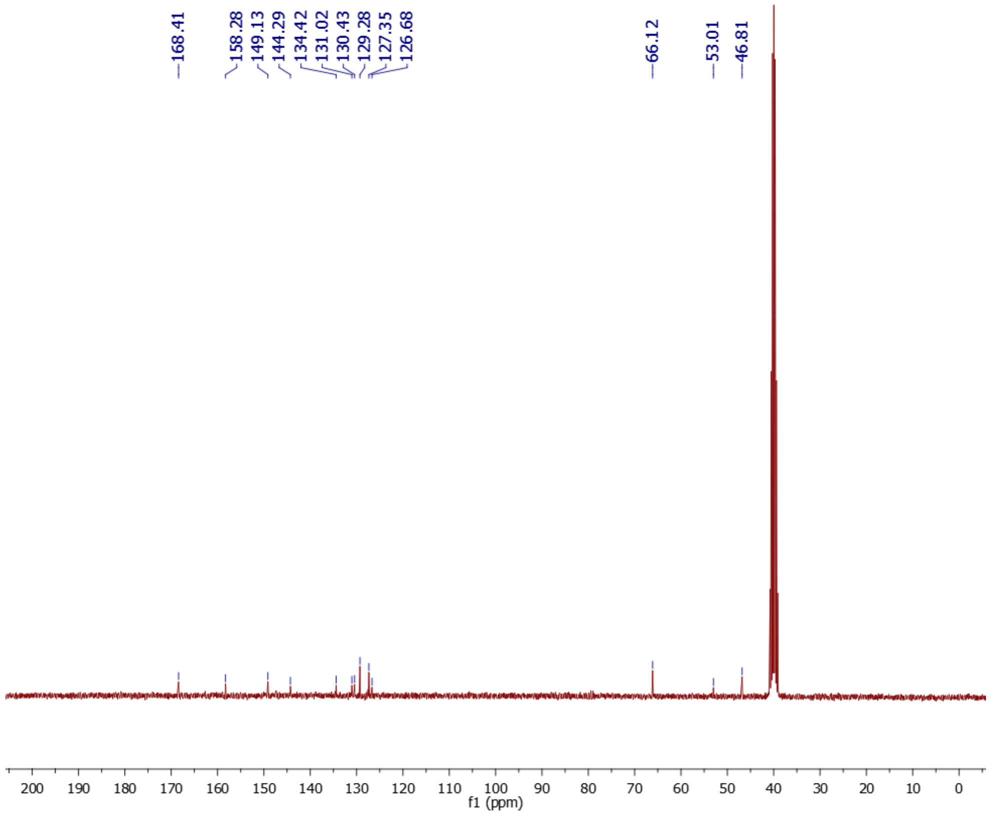

Figure S42. HRMS spectra of compound **TR14**

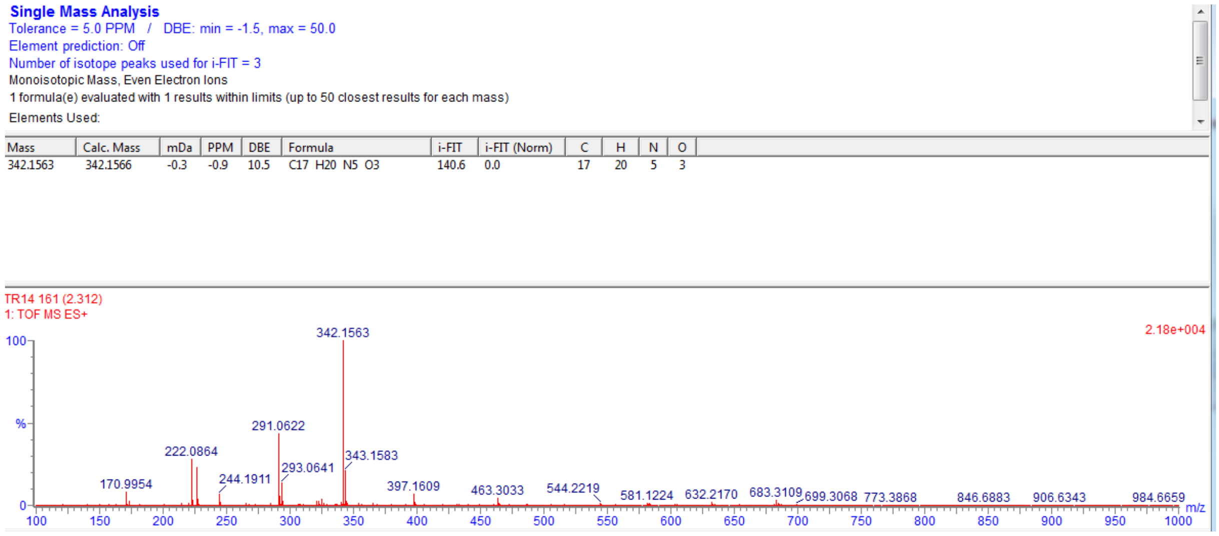

N'-benzylidene-2-(3-(morpholine)-6-oxopyridazin-1(6H)-yl)acetohydrazide (**TR14**)

^{1}H-NMR (DMSO-d$_6$, 300 MHz): δ 3.20 (4H; t; CH$_2$N; b+b'), 3.67 (4H; t; CH$_2$O; a+a'), 5.04 (2H; s; CH$_2$CO), 6.88-7.72 (7H; m; phenyl protons, pyridazinone H^{4,5}), 8.21 (1H; s; -N=CH-) and 11.66 (1H; s; -NH-N).

^{13}C-NMR (DMSO-d$_6$, 300 MHz), δ 46.81 (2C, CH$_2$-N; b+b'), 53.01 (1C; -N-<u>C</u>H$_2$-C=O), 66.12 (2C, CH$_2$-O; a+a'), 126.68 (1C; =CH), 127.35 (1C; phenyl C^4), 129.28 (2C; phenyl C^{3,5}),

130.43 (2C; phenyl $C^{2,6}$), 131.02 (1C; phenyl C^{1}), 134.42 (1C; pyridazinone C^{4}), 144.29 (1C; pyridazinone C^{5}), 149.13 (1C; pyridazinone C^{6}), 158.28 (1C; CH_2-N-C=O), 168.41 (1C; pyridazinone C^{3});

$C_{17}H_{20}N_5O_3$ MS (ESI+) calculated: 342.1566, Found: m/e 342.1563 (M^+; 100.0%).

Figure S43. ^{1}H-NMR spectra of compound **TR15**

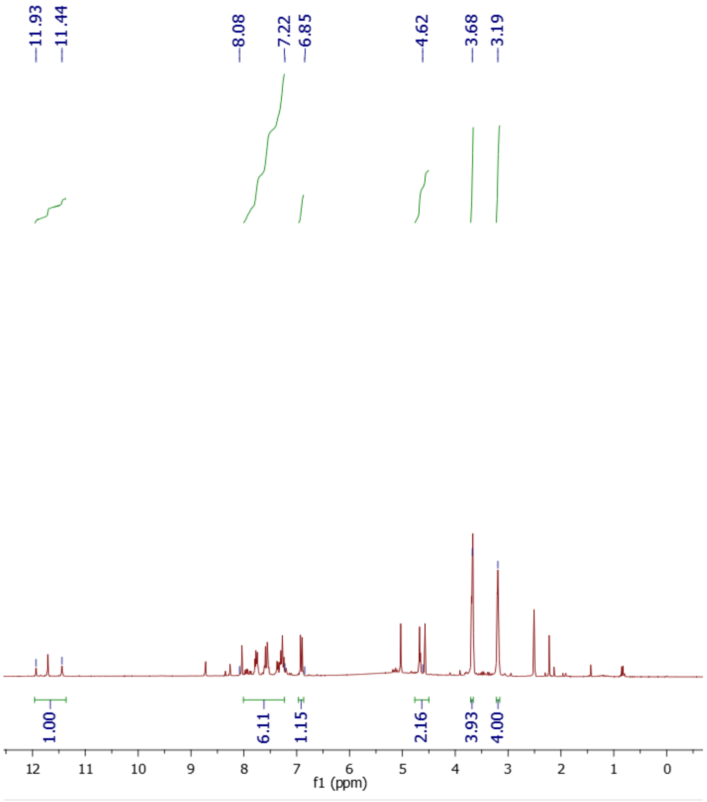

Figure S44. ^{13}C-NMR spectra of compound **TR15**

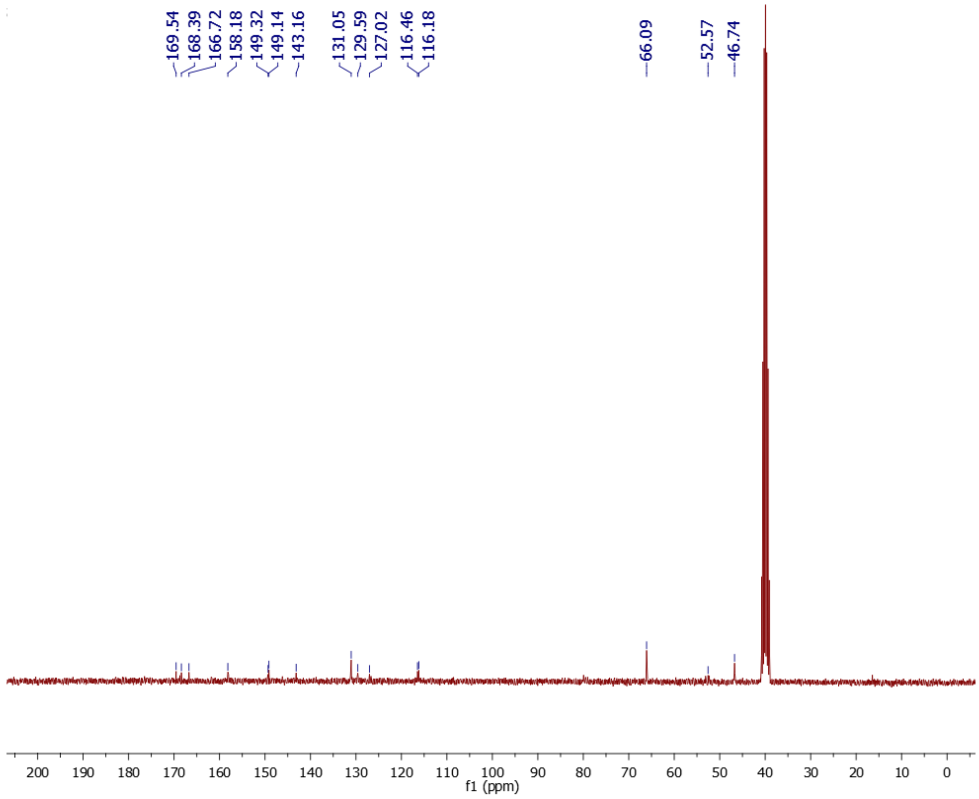

Figure S45. HRMS spectra of compound **TR15**

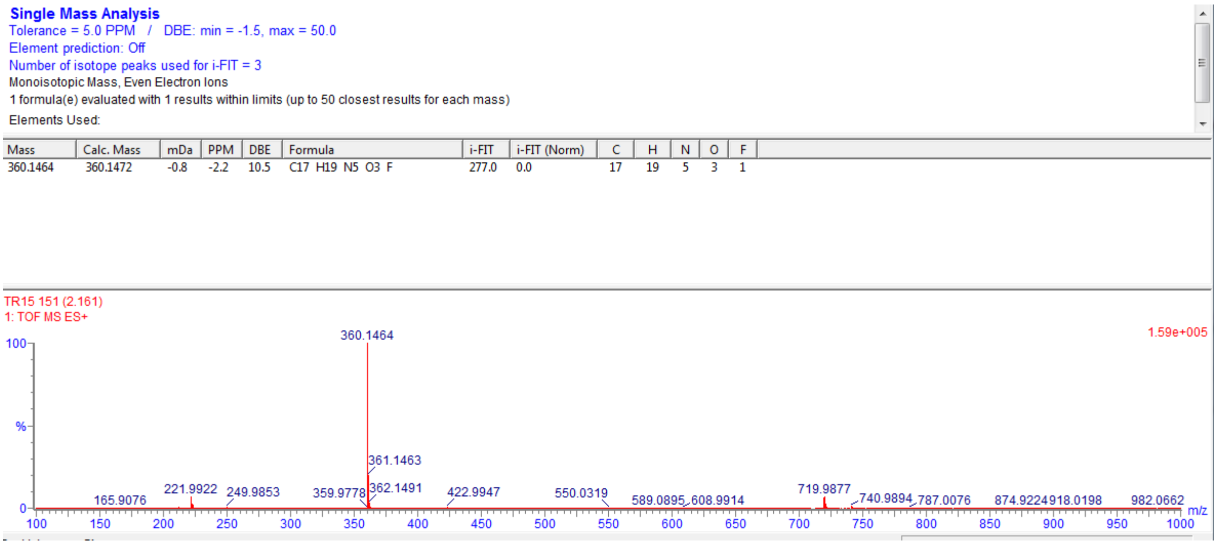

N'-(4-florobenzylidene)-2-(3-(morpholine)-6-oxopyridazin-1(6H)-yl)acetohydrazide (**TR15**)

^{1}H-NMR (DMSO-d_6, 300 MHz): δ 3.19 (4H; t; CH_2N; b+b′), 3.68 (4H; t; CH_2O; a+a′), 4.62 (2H; s; CH_2CO), 6.85-7.22 (7H; m; phenyl protons, pyridazinone $H^{4,5}$), 8.08 (1H; s; -N=CH-) and 11.94 (1H; s; -NH-N).

^{13}C-NMR (DMSO-d$_6$, 300 MHz), δ 46.74 (2C, CH$_2$-N; b+b′), 52.57 (1C; -N-C̲H$_2$-C=O), 66.09 (2C, CH$_2$-O; a+a′), 116.18 (1C; =CH), 116.46 (1C; 4-fluorophenyl C^3),127.02 (1C; 4-fluorophenyl C^5), 129.59 (1C; 4-fluorophenyl C^2), 131.05 (1C; 4-fluorophenyl C^6), 143.16 (1C; 4-fluorophenyl C^1), 149.14 (1C; pyridazinone C^4), 149.32 (1C; pyridazinone C^5), 158.18 (1C; pyridazinone C^6), 166.72 (1C; 4-fluorophenyl C^4), 168.39 (1C; CH$_2$-N-C̲=O), 169.54 (1C; pyridazinone C^3);

$C_{17}H_{19}FN_5O_3$ MS (ESI+) calculated: 360.1472, Found: m/e 360.1464 (M$^+$; 100.0%).

Figure S46. ^{1}H-NMR spectra of compound **TR16**

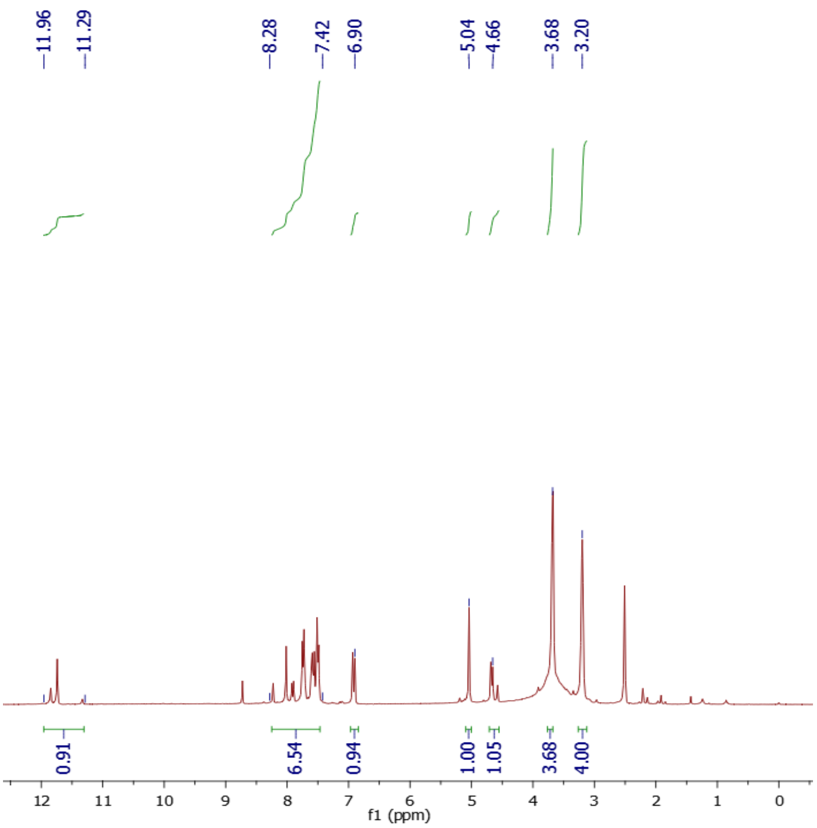

Figure S47. ^{13}C-NMR spectra of compound **TR16**

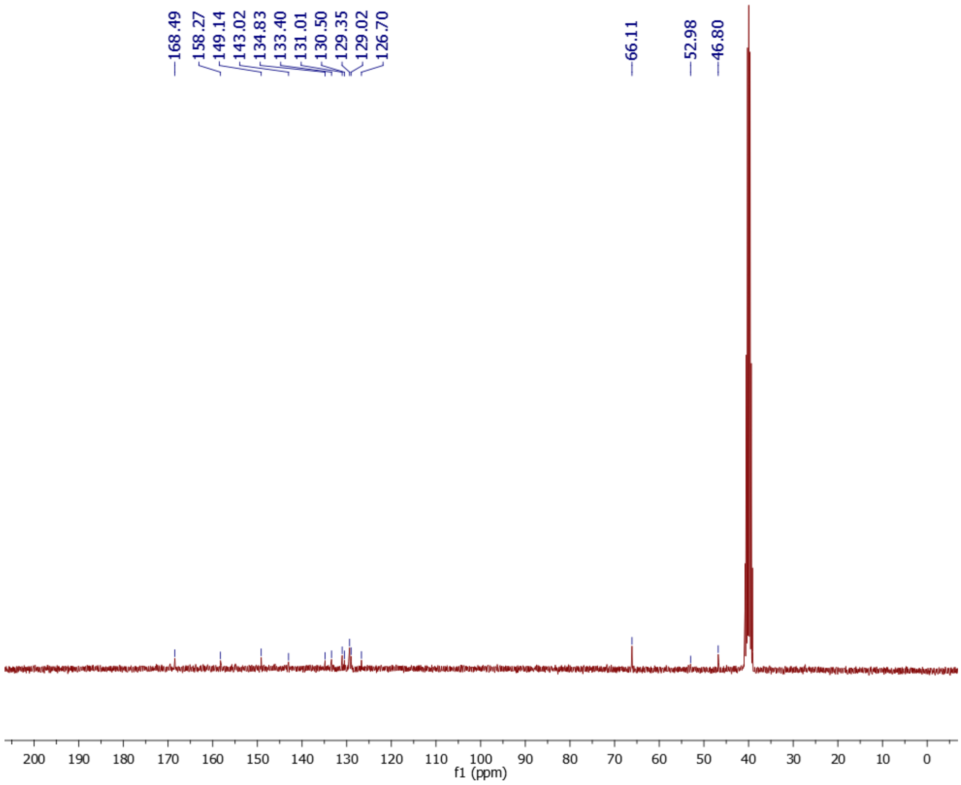

Figure S48. HRMS spectra of compound **TR16**

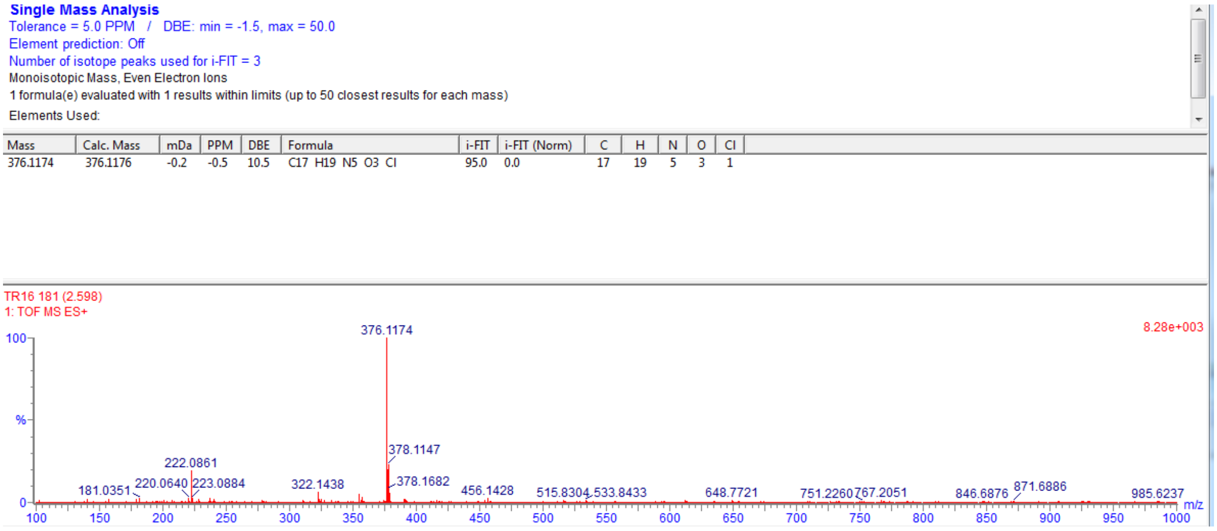

N'-(4-clorobenzylidene)-2-(3-(morpholine)-6-oxopyridazin-1(6H)-yl)acetohydrazide (**TR16**)

^{1}H-NMR (DMSO-d_6, 300 MHz): δ 3.20 (4H; t; CH_2N; b+b’), 3.68 (4H; t; CH_2O; a+a’), 4.66- 5.04 (2H; s; CH_2CO), 6.90-7.42 (9H; m; phenyl protons, pyridazinone $H^{4,5}$), 8.28 (1H; s; -N=CH-) and 11.96 (1H; s; -NH-N).

^{13}C-NMR (DMSO-d_6, 300 MHz), δ 46.80 (2C, CH_2-N; b+b’), 52.98 (1C; -N-$\underline{C}H_2$-C=O), 66.11 (2C, CH_2-O; a+a’), 126.70 (1C; =CH), 129.02 (2C; 4-clorophenyl $C^{3,5}$), 129.35 (1C; 4-

clorophenyl C^2), 130.50 (1C; 4-clorophenyl C^6), 131.01 (1C; 4-clorophenyl C^1), 133.40 (1C; pyridazinone C^4), 134.83 (1C; pyridazinone C^5), 143.02 (1C; pyridazinone C^6), 149.14 (1C; 4-fluorophenyl C^4), 158.27 (1C; CH_2-N-C=O), 168.49 (1C; pyridazinone C^3);

$C_{17}H_{19}ClN_5O_3$ MS (ESI+) calculated: 376.1176, Found: m/e 376.1174 (M^+; 100.0%).

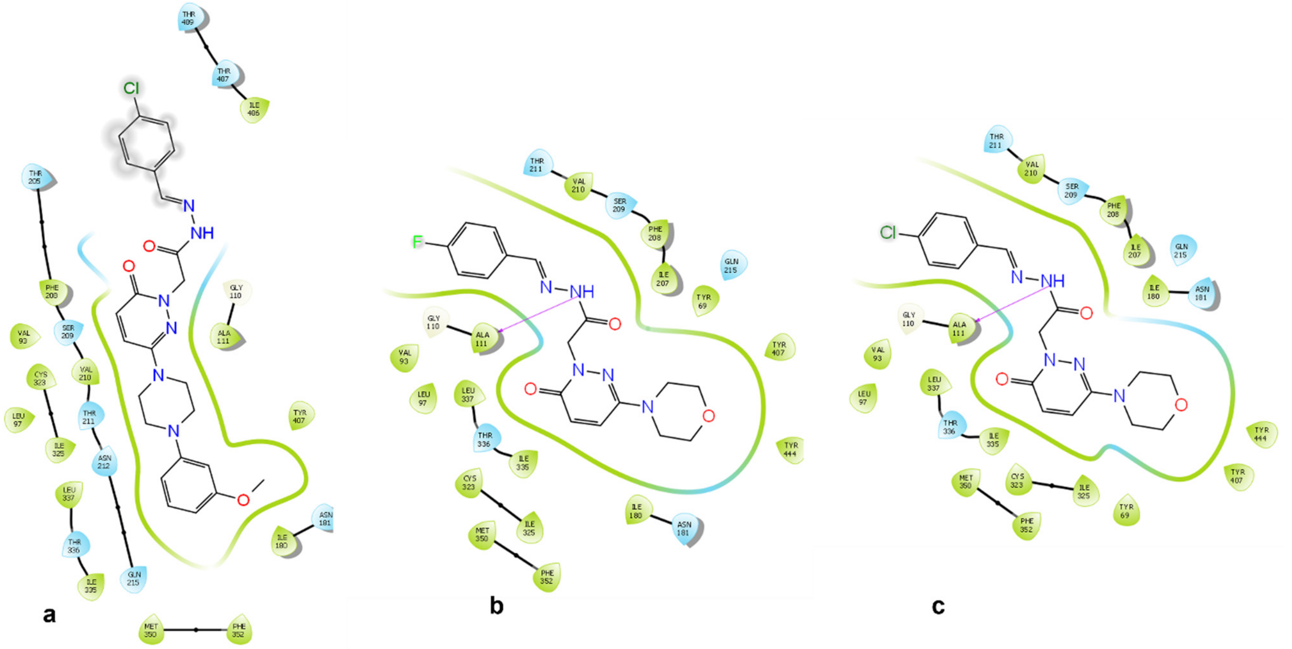

Figure S49. 2D-scheme of interactions of compounds **TR2 (a), TR15 (b),** and **TR16 (c) to** MAO-B showing hydrogen bonds with light blue lines. Ligand exposed to solvent is depicted as grey spheres.

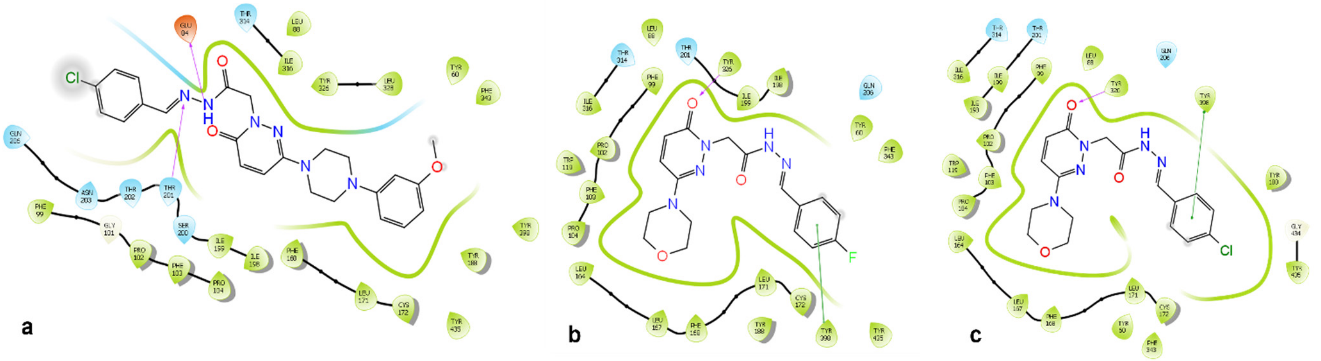

Figure S50. 2D-scheme of interactions of compounds **TR2 (a), TR15 (b),** and **TR16 (c) to** MAO-B showing hydrogen bonds and π-π contacts with light blue and green lines, respectively. Ligand exposed to solvent is depicted as grey spheres.
